# Active bacteriophage biocontrol and therapy on sub-millimeter scales towards removal of unwanted bacteria from foods and microbiomes

**DOI:** 10.3934/microbiol.2017.3.649

**Published:** 2017-08-04

**Authors:** Stephen T. Abedon

**Affiliations:** Department of Microbiology, the Ohio State University, 1680 University Dr., Mansfield, OH 44906, USA

**Keywords:** active treatment, bacteriophage therapy, foodborne pathogens, host range, inundation threshold, microbiome editing, passive treatment, phage therapy, proliferation threshold

## Abstract

Bacteriophages can be used as antibacterial agents as a form of biological control, e.g., such as phage therapy. With active treatment, phages must “actively” produce new virions, *in situ*, to attain “inundative” densities, i.e., sufficient titers to eradicate bacteria over reasonable timeframes. Passive treatment, by contrast, can be accomplished using phages that are bactericidal but incapable of generating new phage virions *in situ* during their interaction with target bacteria. These ideas of active versus passive treatment come from theoretical considerations of phage therapy pharmacology, particularly as developed in terms of phage application to well-mixed cultures consisting of physically unassociated bacteria. Here I extend these concepts to bacteria which instead are physically associated. These are bacteria as found making up cellular arrangements or bacterial microcolonies—collectively, clonal bacterial “clumps”. I consider circumstances where active phage replication would be required to effect desired levels of bacterial clearance, but populations of bacteria nevertheless are insufficiently prevalent to support phage replication to bacteria-inundative densities across environments. Clumped bacteria, however, may still support active treatment at more local, i.e., sub-millimeter, within-clump spatial scales, and potential consequences of this are explored mathematically. Application is to the post-harvest biocontrol of foodborne pathogens, and potentially also to precise microbiome editing. Adequate infection performance by phages in terms of timely burst sizes, that is, other than just adsorption rates and bactericidal activity, thus could be important for treatment effectiveness even if bacterial densities overall are insufficient to support active treatment across environments. Poor phage replication during treatment of even low bacterial numbers, such as given food refrigeration during treatment, consequently could be problematic to biocontrol success. In practical terms, this means that the characterization of phages for such purposes should include their potential to generate new virions under realistic *in situ* conditions across a diversity of potential bacterial targets.

## 1. Introduction

Phage therapy is the use of bacterial viruses, i.e., bacteriophages, as antibacterial agents. This utility dates to the pre-antibiotic, nearly 100-year-old use of phages in the treatment of infectious diseases with bacterial etiologies [1]. Phage therapy today is experiencing a resurgence [2] which stems largely from various negative consequence of antibiotic use, most notably the epidemic rise of bacterial strains and infections which display antibiotic resistance or tolerance [3]. More generally, phage therapy serves as a form of biological control or “biocontrol” of bacteria [4]. Such biocontrol can include the use of phages to combat phytopathogens of crops, the use of phages as disinfectants such as of fomites, or the use of phages to selectively remove undesired bacteria from foods (for a list of reviews of the latter, see the Appendix). In all cases, the first step of successful phage treatment of bacteria is for phage virions which have been added to environments—or which instead have been generated within an environment through phage replication—to encounter bacteria, that is, for virion-bacterium collision to take place. Consideration of the dynamics of phage-bacterial interactions, such as during phage-mediated biocontrol of bacteria, thus has been derived to a substantial extent from that of particle-collision theory.

The basics of particle-collision theory are that particles are free to move through fluids and do so at some rate, plus possess target sizes which are proportional to their radii. The faster the movement, the larger the radius, or the more particles, then the greater the likelihood of collision. Indeed, these ideas can be neatly summarized as (collision frequency) ∝ (particle numbers) × (collision kernel), that is, collision frequency is proportional to the latter product. The collision kernel in turn is simply the product of particle size (i.e., as based on radius) and rate of particle movement, the latter such as rate of diffusion [5].

Particle collisions play important roles in chemical processes and also physical ones. In nuclear chain reactions, for example, fission events are stimulated by the collision of two particles, a fissile atom and a subatomic particle. In this case there are three important differences from that of the more basic theory described in the previous paragraph. The first is that atoms and subatomic particles differ in their respective radii, the second is that their rates of movement also differ, and the third is that in nuclear chain reactions the collision between atom and subatomic particles gives rise to changes in atoms (fission) that results in the production of new subatomic particles. A chain reaction can be self-sustaining when the density of fissile atoms is sufficiently large that the generation of new subatomic particles within systems occurs at least as fast as those subatomic particles are lost from systems. If there are too few fissile atoms present per unit volume for collisions with subatomic particles to be sufficiently likely, if the total volume is too small such that subatomic particles are more likely to be lost from a system than collide with fissile atoms, or if necessary subatomic particles are lost within the system prior to colliding, e.g., as due to neutron “poisoning” by control rods, then the nuclear chain reaction cannot be sustained. The phrase “critical mass” refers to the necessary size of a system to sustain a nuclear chain reaction given some constant density of atoms. More generally, when subatomic particle generation can at least match subatomic particle loss, then a self-sustaining nuclear chain reaction can occur.

These various ideas of collision kernels, particle densities, particle decay rates, and generation of new particles within systems, that is, *in situ*, are all relevant towards appreciating the dynamics of phage-mediated biocontrol of bacteria. In the simplest of systems, we can ignore both particle losses and the generation of new particles. In this case the likelihood of successful bacteria control will be dependent on a combination of particle density and collision kernels, though with two complications shared with nuclear chain reactions. The first is that phages, like subatomic particles, are smaller than their bacterial targets (the latter as equivalent to fissile atoms). The second is that if movement is diffusion limited, then phages, given their smaller size, will be more mobile than bacteria, i.e., as equivalent to subatomic particles versus target atoms. We, however, can ignore phage target size as well as bacterial rate of motion since the collision kernel continues to be a function of target size (now especially the radius of a target bacterium) and rate of movement (now especially the rate of movement, particularly diffusion, of phage particles). As with other processes, there is a less than a 100% likelihood of a given type of interaction between phage and bacterium given collision. Thus we can define a phage adsorption rate constant as equal to this collision kernel (∝ [bacterium radius] × [phage virion diffusion rate]) as multiplied by the likelihood of successful phage adsorption given collision.

Successful biocontrol is dependent on the number of phage adsorptions per bacterium being sufficiently high that some approximation of all bacteria become bactericidally infected. This generally will require relatively high total numbers of phage adsorptions. Increasing numbers of adsorptions per unit time can be accomplished by increasing either the number of phage virions, the number of bacteria, the collision kernel, or the likelihood of adsorption given collisions. Number of collisions overall may be increased also by increasing the length of time over which adsorptions may occur, though over time bacteria may increase in number as well. Allowing bacteria to replicate to higher levels than are already present with systems typically is not desirable, including for reasons of excessive presence of undesired bacteria. So too, for a given phage type, bacterium type, and conditions, the collision kernel and likelihood of phage adsorption given collision with a bacterium will be fixed. Thus, of these variables, the only one which generally is readily manipulated is phage density [6]. Effective phage-mediated biocontrol therefore requires sufficient phage numbers within a given bacteria-containing volume, and particularly sufficient phage numbers as found in the immediate vicinity of target bacteria [7],[8], i.e., just as maintaining a nuclear chain reactions requires sufficient atom-colliding subatomic particles.

With phage therapy or phage-mediated biocontrol, as also with nuclear chain reactions, there are two ways of supplying these smaller, faster particles to systems, and this is either by adding them from outside of the system or instead by generating them within the system. Confusingly [9], we describe phage therapy which is motivated solely by the supplying of phages from outside of systems as “passive”. By contrast, the supplying of phages from some combination of external and internal sources we describe instead as “active”. (Phages which are already present within environments instead are “endogenous” [10].) Here internal sources are a consequence not of nuclear fission but instead of phage-productive infections of bacteria, that is, resulting in the generation of new, small, relatively rapidly diffusing particles, i.e., new phage virions. Active treatment thus can be viewed as the phage-mediated biocontrol equivalent of a self-sustaining chain reaction. A glossary of various terms and concepts relevant to this study can be found in Table 1.

**Table 1.** Defining key concepts.

Concepts	Definitions and Discussions
Clump	Physically associated, clonally related bacteria such as making up cellular arrangements or microcolonies
Across environments	Equivalent to “global” or “globally”, with environment referring to the area or volume which has been subject to phage treatment; here this phrase is used to indicate an attainment of inundative phage densities that occurs effectively everywhere within a given, treated environment, e.g., such as across a single phage-treated food item
Global	Referring here to the entirety of a phage-treated environment
Local	Referring here to a subset of an environment roughly on the scale of a bacterial clump and generally corresponding to distances which are somewhat less than 1 millimeter
Active treatment	Phage therapy or phage-mediated biocontrol of bacteria where phage multiplication *in situ* is required to achieve “inundative” phage densities
Active adsorption	Bacterial adsorption as achieved by phages which have been generated *in situ* through phage population growth
Globally active treatment	Active treatment where inundative phage densities are achieved through phage replication across the totality of an environment, , e.g., >>1 mm spatial scales, versus only locally
Locally active treatment	Active treatment which is accomplished only locally within environments; though globally active treatment may not be achieved in a given instance due to insufficient overall (i.e., global) densities of target bacteria, locally active treatment may still be achieved given sufficient bacterial densities over sub-millimeter scales, i.e., as due to bacterial clumping
Passive treatment	Phage therapy or phage-mediated biocontrol of bacteria where phage multiplication *in situ* is not required to achieve inundative phage densities; with obligately passive treatment such *in situ* phage multiplication is not even possible; here the qualifier “purely” is used to emphasize that only passive treatment is being considered in a given situation
Passive adsorption	Bacterial adsorption as achieved by phages which are supplied to environments via dosing
Inundation therapy	Passive treatment
Inundative densities	Sufficient phage densities to achieve not just some reasonable approximation of elimination of target bacteria, during phage therapy or phage-mediated bacterial biocontrol, but elimination over reasonable time scales, e.g., minutes or hours rather than weeks or months
Threshold bacterial density	Sufficient bacterial densities to support the maintenance of a phage population where that phage population otherwise would be faced with decay in numbers of virions
Proliferation threshold	Equivalent to threshold bacterial density
Inundation threshold	Sufficient densities of phage virions, holding phage densities constant, to result in declines in numbers of sensitive replicating bacteria over time; *inundative* phage densities, by contrast and by definition, must not only exceed inundation thresholds for net bacterial elimination to occur but generally must exceed those thresholds by a substantial amount since otherwise desired elimination of bacteria will occur at too slow a pace
Eradication threshold	Sufficient phage densities to result in declines in numbers of sensitive replicating bacteria over time, assuming that locally active treatment is possible, i.e., as representing an inundation threshold that is applicable to the “eradication” of bacterial clumps versus as applicable solely to the “inundation” of individual bacteria
MIC	Minimum Inhibitory Concentration, a concept which is not easily applied to phages
MEC	Minimum Effective Concentration; with phages MEC generally is either less than or equal to MIC and as with MIC, MEC generally is an *in situ*, post-dosing antibacterial density

The basic theory of passive and active treatments has been worked out [11],[12],[13]; see also [9],[14],[15],[16]. This theory is as applied to systems in which bacteria are homogenously present across considered environments and also in which both phages and bacteria are well mixed within those environments, that is, are unconstrained in terms of the rate that any given phage can find any given bacterium. These assumptions simplify the underlying mathematics but are not necessarily fully applicable to environments which are neither fully homogeneous, such as with regard to bacterial concentrations, nor well mixed. Importantly, one key exception is phage interaction with bacteria which are not isolated from one another but which instead are found associated into numerous clonal “clumps” [3],[17]. Here I provide extensions of the theory of phage-bacterial interaction dynamics as applicable to the phage-mediated biological control of bacteria, with undesired bacteria existing as clonal clumps rather than being present as well-mixed cultures of individual, isolated cells.

Clumps can consist of cellular arrangements such as streptococci, cells which are associated as a consequence of a failure to physically move apart following cell division (microcolonies), or indeed not-so micro colonies as well as single-species biofilms. Clumps furthermore may be assumed to be of increasing size given greater numbers of constituent bacteria per clump. Considered here is the potential for clumped versus not clumped bacteria to support phage population growth to inundative densities (Sections 3.1. and 3.2.) along with changes in the susceptibility of bacterial populations to phages as could result from bacterial presence within clonal clumps (Sections 3.3., 3.4., and 3.5.). The terms “global” and “local” are used here extensively and these refer to different spatial scales regarding the “systems” which are under consideration, e.g., a single batch of food or a single infection such as of an animal. “Global” thus refers to across the totality of the system under consideration whereas “local” refers to a small volume, typically a subset of a larger system. In particular, here “local” refers especially to spatial scales which are on the order of the size of clonal bacterial clumps, especially sub-millimeter lengths, whereas “global” refers to spatial scales which are much larger, especially somewhat greater than millimeter lengths. The phrase “across an environment” (environment as a single system) and “global” are thus used equivalently.

Four major points are argued. (I) Active treatment as usually defined—that is, a globally active treatment—is rarely expected to play a role in phage-mediated biocontrol of bacteria which contaminate foods, as densities of problematic bacteria in still-comestible foods generally are insufficiently high [6],[18] to globally support phage population growth to inundative densities; (II) Given bacterial clumping, then *in situ* phage replication to higher densities still nonetheless could occur but only highly locally (<<1 millimeter spatial scales), that is, as resulting in a locally active treatment, and such locally active treatment may be important to phage-mediated biocontrol of bacteria within foods; (III) For the sake of more robustly reducing densities of problematic bacteria in foods and/or supplying extra margins of antibacterial effectiveness, then using phages which are able to replicate under expected treatment conditions, thereby contributing to a locally active treatment given bacterial clumping, could be preferable to using phages which are unable to replicate under prevailing treatment conditions, e.g., as due to refrigeration; (IV) As a consequence of this latter point, during phage host-range determination the potential for phages to productively infect, i.e., produce virions while infecting a diversity of target bacteria under anticipated *in situ* conditions, should be explicitly assessed. To reiterate, (I) we have reason to expect that phage *in situ* replication could be irrelevant to biocontrol of bacteria within foods; (II) given bacterial clumping this expectation in fact may not be entirely correct; (III) there therefore could be a utility to treating foods using phages which are able to replicate in the course of those treatments, and (IV) efforts should be made to use such phages as warranted.

An important aspect of using phages in foods, or indeed as antibacterial agents generally, is that, properly chosen, phage-mediated biocontrol tends to result in few negative impacts, e.g., such as on our health. This safety has been demonstrated clinically in a number of phase I trials [19]–[26], but also as indicated by the Generally Recognized as Safe (GRAS) designation for phage use in foods [27]. Given such potential safety, an additional circumstance besides biocontrol within foods in which target bacteria could support locally active treatment, but not necessarily globally active treatment, is the selective elimination of problematic but nonetheless minor components of spatially structured microbiomes, such as those microbiomes associated with our bodies. That is, modification of microbiomes by an “editing out” of unwanted bacteria, with biocontrol of specific bacteria in foods also serving as a form of food-microbiome editing. Unlike the phage treatment of what are more obviously pathogens, it can be mostly healthy individuals who would be subject to microbiome editing. Though safety therefore is certainly important, it nonetheless is the high specificity of phages for bacterial targets which is key to their proposed use towards highly selective microbiome editing. Here, again, using phages which explicitly are able to productively infect target bacteria under *in situ* conditions could be helpful, even if overall densities of these bacteria are insufficient to support globally active treatment. Thus, as with active treatment more generally, a premium should be put on making an effort during *in vitro* phage characterization to determine phage host ranges other than solely in terms of bactericidal effects [28],[29].

## 2. Materials and Methods

Existing equations describing various aspects of phage-therapy pharmacology are modified to take into account the greater overall target size as well as greater number of bacterial cells as associated with clonal bacterial clumps, especially microcolonies or cellular arrangements, versus individual, spatially isolated bacteria. A primary assumption made in these modifications is that the environments containing these bacterial clumps are not highly mixed such that phage movement to as well as away from these clumps is a function primarily or entirely of virion diffusion. The terms “global” and “globally” refer to across phage-treated environments, e.g., an entire batch of phage-treated food, whereas “local” and “locally” refer to as within the immediate vicinity of a bacterial clump, i.e., as over sub-millimeter scales. Unless explicitly indicated otherwise, bacterial densities as well as the densities of phage-virions are assumed to remain constant over time, especially globally. Exceptions are seen when considering phage-virion inactivation (where phage densities can decline) along with times (durations) over which bacteria are reduced in density given phage exposure (where bacterial densities decline rather than remaining constant).

## 3. Results

### 3.1. Inundative phage densities

For phage-mediated biocontrol to be effective in eliminating phage-sensitive target bacteria, then phages must attain sufficient densities (titers) within the vicinity of those bacteria to result in some approximation of 100% of them becoming phage adsorbed. Typically not only are substantial degrees of adsorption of targeted bacteria required, meaning multiple phage adsorptions to each targeted bacterium [15], but it is desirable that this adsorption occur over reasonable time frames, e.g., minutes or perhaps hours rather than weeks, months, or years [30]. Here the term “inundative” is used to describe phage densities which are capable of achieving these levels of timely killing of populations of targeted bacteria. Note, for the sake of avoiding confusion, that no assumption is being made about where or when this bacterial killing occurs, e.g., such as only upon warming of refrigerated foods, just that it occurs post phage adsorption.

Except for very high bacterial densities (e.g., ≥10^7^ bacteria/ml), then a fair approximation of what constitutes inundative for a typical combination of phages and bacterial targets is 10^8^ phages/ml, or greater [6],[7],[16],[18] (see also the Appendix); for per cm^2^ units rather than per ml, see [31]. Inundative phage densities in phage-mediated biocontrol of bacteria may be achieved either solely via traditional dosing, which is termed passive treatment, or achieved via a combination of traditional dosing and *in situ* phage replication, i.e., active treatment [11],[12],[13].

It is important to appreciate, for reasons explained in Section 3.1.2., that densities of target bacteria as found in foods subject to phage-mediated biocontrol (Section 4.2. as well as the Appendix) generally are insufficient to support phage population growth to inundative densities across environments, i.e., such as globally across a treated food. What this means in practice is that phage titers generally cannot rise to, for example, 10^8^/ml when only 10^4^ bacteria/ml are present within an environment (ignoring complications associated with converting to per gram or per cm^2^ units). Therefore, it should be assumed that passive treatment, to at least a first approximation, must be employed in such biocontrol, or indeed employed in any circumstance in which densities of target bacteria are insufficiently high to support phage population growth to inundative densities. In other words, with typical phage treatment of foods it is necessary to supply via dosing all phages required to attain inundative densities rather than relying on *in situ* phage population growth to make up for deficiencies. It may not be entirely correct that biocontrol of foods is limited to such purely passive treatment under all circumstances, however. This latter issue, in turn, is the primary consideration of this article.

Note, for the sake of consistency, that I employ the terms “passive” and “active” also in describing the source of phage adsorptions during treatment. Adsorptions which are “passive” thus are by virions that are supplied directly by dosing. Adsorptions which are “active” instead are by virions which have been generated *in situ* via phage replication. That is, passive treatment gives rise to passive adsorptions while active treatment is associated initially with passive adsorptions (giving rise to epidemiologically primary phage infections [32]), but subsequently with active adsorptions (giving rise, epidemiologically speaking, to secondary infections). Regardless of the treatment strategy employed, passive versus active, successful removal of bacteria from environments requires the achievement of inundative phage densities. Here, an additional distinction is introduced regarding active treatment, that of active treatment over larger spatial specials (globally active treatment) versus active treatment as may be observed over only smaller special scales (locally active treatment). Inundative densities reached, for example, over the entirety of a phage-treated environment, i.e., especially over greater than millimeter scales, would be an example of globally active treatment whereas inundative phage densities achieved over much smaller spatial scales, i.e., sub-millimeter, would be an example of locally active treatment. See Table 2 for summary as well as further exploration of these various concepts.

**Table 2.** Comparing concepts.

	Passive Treatment	Globally Active Treatment	Locally Active Treatment
Means by which inundative densities^1^ are achieved	Sufficient phage numbers to eradicate target populations of bacteria are supplied via dosing alone	Sufficient phage numbers across environments are generated *in situ* via phage replication	Sufficient phage numbers are generated *in situ* over especially local scales, e.g., <<1 millimeter
Requires *in situ* phage replication	No	Yes	Yes
Requires phage movement between bacteria	No	Yes	Only within bacterial clumps rather than between clumps
Can require substantial increases in phage densities across environments	No (but could involve such increases)	Yes	No (but could involve such increases)
Can be effected by bactericidal but not replication competent phages	Yes	No	No
Requires environmental inhomogeneities, i.e., poor mixing	No	No	Yes
Requires inundative phage densities in order to be effective	Yes	Yes	Yes (at least locally)
Requires dosing with minimally effective phage concentrations^2^ (MECs)	Yes	Yes	Yes
Inundative phage densities (IPDs) are equal to MECs	Yes	No (MECs < IPDs)	No (MECs < IPDs)
Involves passive adsorption^3^	Yes	Yes	Yes
Requires active adsorption^4^	No	Yes	Yes
Concept also known as	Inundative Treatment	Active Treatment	Spatial Vulnerability
References	[11],[12],[13]	[11],[12],[13]	[33]

^1^ Inundative phage densities are those required to achieve eradication of targeted bacteria over desired time scales. ^2^ Minimum effective phage densities (MECs) are those, as achieved solely via dosing, which are sufficient, ultimately, to achieve inundative phage densities, i.e., these either are equal to inundative phage densities, for successful passive treatment, or instead can give rise to sufficient *in situ* phage population growth to achieve inundative densities (active treatment). ^3^ Passive adsorption is the attachment of virions, as supplied solely by dosing, to target bacteria, giving rise as viewed from an epidemiological perspective to what are known as primary infections. ^4^ Active adsorption is the attachment of virions, which have been generated *in situ* in the course of phage replication, to new target bacteria, giving rise as viewed from an epidemiological perspective to what are known as secondary infections.

#### 3.1.1. Basic theory of inundation

The rate at which individual bacteria are found by phages is a function, minimally, of the density of free phages within environments (*P*; a.k.a., *in situ* phage titer) along with the phage adsorption rate constant (*k*). The latter combines the collision kernel with the likelihood of phage adsorption given collision with a bacterium (Section 1.). This rate is equal to simply *Pk*. What this expression tells us is that, all else held constant, bacteria will be adsorbed by phages at a faster rate the more phage virions which are present within an environment. The greater the peak in phage density within an environment, and the longer this peak or something close to this peak can be sustained, then the fewer bacteria—ones which are sensitive to these phages—should persist unadsorbed over time.

With 10^8^ phages/ml, and a phage adsorption rate constant of 2.5 × 10^−9^ ml/min [34], then after 60 min an average of 15 phages will have adsorbed per bacterium. Note that this claim assumes that phage densities remain constant over this period, an assumption that is less likely to be violated given lower initial densities of target bacteria, and is based on the expression, *Pkt*, where *t* is time. This multiplicity of infection (MOI), or more specifically what can be described as an MOI_actual_ [35], corresponds—assuming that phages adsorb to bacteria Poissonally [36]—to a bacterial survival rate of e^−15^, or less than 1 in 1 million (actually, 3.1 × 10^−7^; note that “e” is the base of the natural logarithm). This is the predicted frequency of not-phage-adsorbed bacteria after the indicated one hour of adsorption, assuming 100% of targeted bacteria are equivalently susceptible to phage adsorption.

This degree of bacteria killing may literally represent “overkill”. Nonetheless, within- or between-population variation in bacterial susceptibility to phage adsorption (i.e., slower rates than as assumed here), phage difficulties in reaching targeted bacteria (such as due to the presence of virion-diffusion inhibiting food matrix), or simply time constraints may imply a utility to at least striving for such low levels of bacterial survival. This idea of supplying sufficiently high phage densities to achieve overkill of targeted bacteria is further addressed in the Appendix.

If faster phage adsorption or greater margins of antibacterial effectiveness are desired, then more phages will be required. With 10^9^ phages/ml, using the same parameters as above, then well over one billion-fold killing can occur given 10 min of adsorption. Therefore, though the concept of inundative phage densities may be defined in various ways depending upon time constraints along with the extent to which one hopes to reduce bacterial populations in number, a reasonable phage inundative density is not likely to be much less than 10^8^ phages/ml, and potentially more phages will be required. As considered in the Appendix, and here [37], an appreciation of whether such phage densities in fact are reached in the course of biocontrol at a minimum requires knowledge of phage titers as found within applied formulated products.

#### 3.1.2. Achieving inundative densities

Given purely passive treatment, that is, with all phages which are present within an environment supplied via dosing, then peak phage densities along with their persistence *in situ* can be achieved more or less independent of conditions within those environments, i.e., simply by adding more phages as needed. This should remain true so long as phages may be readily delivered directly to targeted bacteria. Purely passive treatment, however, can be undesirable for economic, safety, or even convenience reasons. Undesirables, for example, can include requirements for spraying over vast areas in agriculture, given concerns over excessively rapid bacterial lysis, desires not to introduce excessively large numbers of virion particles into animals, or in terms of difficulties associated with repeated dosing regimens as can be required to maintain phage densities at high levels via purely passive means over long periods.

With active treatment, by contrast, then phage densities in theory can be maintained at necessarily high levels not through high-level or ongoing phage dosing but instead as a consequence of an essentially as-needed phage replication in association with target bacteria. Though a desirable, almost homeostatic process, with active treatment then peak phage densities as well as the persistence of these densities becomes highly dependent on both phage and environment properties, specifically in terms of the number of bacteria present, their potential to support phage replication, and the ability of phages to use this replication to substantially boost their *in situ* numbers. In particular, bacteria may support phage replication up to a maximum (peak) which approaches *NB*, where *N* is bacterial density and *B* is phage burst size as observed *in situ*. The latter actually is no more than *B* – 1 to account for phage losses due to infection-initiating phage adsorptions, but for simplicity's sake net increases in phage numbers of one burst size per phage infection will be assumed.

Assuming (i) that 10^8^ phages/ml represents an inundative phage density, (ii) phage burst sizes of at least 100, and (iii) fairly fast phage adsorption to individual bacteria, then a minimum of approximately 10^6^ bacteria/ml will be required to support active treatment (i.e., 100 × 10^6^ = 10^8^). Thus, unless the bacteria being treated are present within environments at fairly high densities, certainly higher than would be preferred in foods in terms of foodborne pathogens, then it is unlikely that successful active treatment may be accomplished. That does not mean that bacteria at lower densities will not be able to support net phage population growth, but instead that at lower bacterial densities such growth will not be to inundative densities across environments. It is important to recognize, however, that an important underlying assumption to this statement is that these calculations are based both on substantial environment mixing and bacteria not existing within clonal clumps. What, then, may be the consequence of revising these assumptions? We start especially with the question of whether bacterial existence within clonal clumps might impact the ability of phages to reach inundative densities, but then quickly move on to the issue of environmental mixing.

#### 3.1.3. Factoring in bacterial existence within clonal clumps

What may be the consequence of bacteria presence within clonal clumps on the potential for those bacteria to support phage replication to inundative densities? There are at least three possible answers to this question: (a) no difference, (b) reduced potential, and (c) greater potential. The “no difference” answer (a) takes into account only the number of bacteria present per unit volume, that is, *N*, which is assumed throughout this study to not change across environments when going from not-clumped to clumped bacteria. If these bacteria are all readily reached by phage virions and all bacteria are equally capable of supporting equivalent burst sizes (*B*), then the hypothetical maximum for peak phage density, defined as *NB*, should not vary as a function of bacterial clumping. Furthermore, if we do not exclude environmental mixing, then those phage densities, whatever they may be, may be achieved across environments.

The second answer (b), “reduced potential” for clumped bacteria to support phage replication to inundative densities, assumes that at least one of these conditions does not hold given bacterial clumping, e.g., such as there being physiological differences between bacteria, particularly some bacteria displaying more stationary-phase like physiologies, or instead because of barriers to phage access to at least some bacteria, in this case barriers which are a direct consequence of bacterial clumping. Given one or both conditions, then peak phage densities may be lower due to reduced bacterial ability to support substantial phage burst sizes [38] (smaller *B*) or instead due to shading of bacteria by each other [39],[40],[41],[42], the latter arguably an example of masking of receptors [43]. Shading results in lowered phage accessibility to bacteria [8],[33] and thus at least potentially leads to an effectively smaller *N* (cell density). Given these scenarios, (a) and (b), then we would conclude that peak phage densities across environments are unlikely to be greater and in fact could be lower given bacterial clumping. From this we would conclude, in terms of the generation of peak phage densities, that there should be either no difference in the ability of phages to impact target bacteria as a function of clumping (a), or instead a reduced phage impact (b).

For the third answer (c), “greater potential” for clumped bacteria to support phage replication to inundative densities, no additional assumptions are being made except that environments are not necessarily extremely well mixed. Absent effective mixing, that is, with movement within environments largely limited especially to diffusion, then phage densities may vary across environments. In particular, phage densities should be greater in the immediate vicinity of lysing bacteria versus elsewhere within environments. The result, given bacteria presence within clonal clumps, should be greater phage densities in the vicinity of clumps which contain lysing bacteria versus in the vicinity of more distant bacteria or as associated with clumps of bacteria which do not contain lysing bacteria. That is, as a consequence of a combination of active phage replication, associated virion release, and virion movement which occurs via diffusion alone, then peak phage densities within the immediate vicinity of target bacteria should be greater for a locally higher density of bacteria, given phage infection of those bacteria, than may be achieved over the entire span of the same environments. This impact of clumping in fact can be positive (c, greater potential) immediately locally even if more globally it is negative (b, reduced potential). That is, even if some phage bursts are reduced in size as a consequence of clumping, or some bacteria are less readily reached, there still can be higher phage densities immediately local to physically adjacent bacteria versus across larger environments.

I have considered in detail elsewhere [8],[33] this idea of localized phage population growth in association with clonal bacterial clumps, increased localized peak phage densities (which ideally, for phage treatment, is to inundative densities), and resulting increased localized phage impact on bacteria. Here the goal is to explore the consequences of these assumptions on phage impact on bacteria especially as during phage-mediated biological control of bacteria such as in foods, and particularly in comparison to phage impact absent bacteria presence within clonal clumps. First, though, I take a more formal look at the potential for bacteria to support phage replication to higher, especially inundative densities. This is done both with and without bacterial clumping and in terms of highly local (e.g., <<1 millimeter scales) versus more global effects.

### 3.2. Threshold bacterial density towards sustaining phage populations

The key to successful active treatment is for bacteria to be present within environments at concentrations which are greater than what Payne et al. [11],[12],[13] described as a proliferation threshold (or “proliferation density threshold”). A proliferation threshold is that density of bacteria which is required to exactly balance—via phage infection and subsequent replication—the rate that phages will decrease in number due to virion inactivation. Here bacterial density is a key variable because the rate that phages adsorb is proportional to that density. Thus, the more bacteria that are present per unit volume then the more likely phages will adsorb per unit time, all else held constant, and thereby not become inactivated as virions. Phage adsorption results in phage infection and, subsequently, the release of a burst of new phages. A new phage virion that is released from a bacterium, however, is subject to a risk of inactivation at some constant rate, i.e., as equivalent to neutrons being poisoned within or otherwise escaping from a mass of fissile material (Section 1.). The proliferation threshold thus is a “critical density” in which fast moving particles are lost within a system as fast as they are generated whereas with a “critical mass” the fast moving particles are lost especially by escaping from the system altogether. Otherwise, however, the two concepts are quite similar.

The proliferation threshold is that bacterial density at which, on average, all of a phage burst becomes inactivated prior to adsorbing, except for a single virion which succeeds in productively infecting. For active treatment to occur, however, then by definition on average more than one phage per burst must survive to productively infect new bacteria. The questions posed immediately above (Section 3.1.3.) therefore can be restated, at least in part, in terms of whether proliferation thresholds as measured in terms of densities of individual bacteria will change as a consequence of bacterial clumping. That is, given clumping, will fewer bacteria per unit volume across environments be required to reach the proliferation threshold, more, or just the same? The answers, also as equivalent to as indicated above, are at best possibly fewer bacteria will be required more globally given clumping—possibly answer (c), greater potential, but perhaps instead answer (a), no change, or maybe even answer (b), reduced potential (Section 3.1.3.)—but also that proliferation thresholds may be readily exceeded over spatial scales which are closer to the size of bacterial clumps, i.e., <<1 millimeter; answer (c) (Section 3.1.3). Keep in mind that *N*, as above, is the actual density of bacteria within environments and that this value is being held constant in comparing scenarios. That is, bacterial existence within clonal clumps does not have an effect of increasing the total number of bacteria found within an environment, but rather just their distribution, clumped versus not.

#### 3.2.1. Proliferation threshold without clumping

Proliferation threshold I abbreviate as *N*_T_, where is *N* is bacterial density and T refers to Threshold. *N*_T_ thus refers to that minimum bacterial density required to sustain a phage population assuming some rate of phage virion inactivation, *X*. By contrast, if there were no virion loss then any bacterial density greater than zero would sustain phage population growth. Rates of phage inactivation may not be substantial and also, operationally, may be a consequence of phages departing from the vicinity of target bacteria rather than becoming outright inactivated (re: critical mass, Section 1.). In any case, this calculation can provide some sense of what bacterial densities are necessary to at least sustain phage populations over time, though as noted the goal with active treatment instead is typically to achieve substantial increases in phage densities over time rather than merely sustaining a phage population at some minimal level (Section 3.1.2.). To achieve substantial increases in phage densities over reasonable time frames, however, then bacterial densities which are substantially greater than *N*_T_ generally will be required. In other words, proliferation thresholds are a necessary but nevertheless not sufficient condition towards attaining inundative phage densities via active phage replication *in situ*. It therefore is never reasonable to simply assume that an acceptable active treatment outcome will result as a consequence solely of bacteria simply exceeding this proliferation threshold, though certainly successful active treatment will *not* result if bacterial densities (at all spatial scales) are found at or below this threshold.

Notwithstanding that *N*_T_ at best defines the absolute minimum density of bacteria only above which active treatment becomes even remotely possible, we can derive *N*_T_ starting with the following equation:

$$\frac{dP}{dt}=BPkN-PX.$$ (1)

Here *P* is phage virion density, *B* the phage burst size, *k* the phage adsorption rate constant, *N* bacterial density, and *X* the rate of phage virion inactivation. In words, this equation indicates that rates of change in phage densities (*dP*/*dt*) are equal to rates of *in situ* phage production (*BPkN*) minus rates of virion inactivation (*PX*). Since we are interested in that threshold at which phage densities are held constant despite ongoing phage replication along with virion inactivation, while also holding bacterial densities constant, we therefore can set the equation equal to zero, meaning no instantaneous change in phage densities over time.

Setting the above equation equal to zero, *N* equal to *N*_T_, and then rearranging, we therefore have,

$$N_{\text{T}}=X/Bk,$$ (2)

which defines the proliferation threshold. Phages thus must increase their numbers on a per-capita basis (*BkN*, i.e., burst size multiplied times the likelihood of an individual phage adsorbing) at least as fast as individual phage virions are inactivated, at rate *X*, in order for phage population densities to remain constant (i.e., such that *dP/dt* = 0), and this balance occurs with a bacterial density as defined in the above equation.

Note that an alternative derivation of this equation, as simplified from that of Payne and colleagues [11],[12], and also ignoring bacterial population growth, is provided by Abedon and Thomas-Abedon [9]. Notwithstanding the existence of multiple approaches to defining or deriving this proliferation threshold, the question posed here is whether *N*_T_ will change in magnitude given bacterial existence within clumps rather than as isolated cells. This question is addressed in the following two subsections (3.2.2 and 3.2.3). The answers generally will be perhaps not (answer a, “no difference”, Section 3.1.3) and likely (answer c, “greater potential”), corresponding to globally active treatment versus locally active treatment, respectively.

#### 3.2.2. Proliferation threshold with clumping

To apply the concept of a proliferation threshold to bacteria which are not randomly distributed across environments, but which instead are present within clonal clumps, we need to add an additional parameter, *n*. This, at least simplistically, is the number of bacteria present per clump which will become infected, and thereby which individually will generate a burst size of *B* every time a clump has become phage adsorbed (and thereby, as a clump, is no longer phage free). In other words, the assumption is that a single phage adsorption to a bacterial clump will result in sufficient phage population growth immediately local to that clump that all other bacteria making up the clump will become phage infected and thereby also individually produce a phage burst sizes of *B*. To keep things simple, we assume that clumps are initially adsorbed, that is, passively adsorbed (Section 3.1.), by only a single phage at most. That is, across the bacterial population, rates that phages passively adsorb are low on a per-bacterium basis, meaning especially that *Pk*, the rate per unit time and unit volume that bacteria are adsorbed by phages, is small relative to *N*/*n*, which is the density of bacterial clumps. Note that the effect of relaxing this assumption would be to reduce the effective magnitude of *n*, an issue which in various ways is addressed subsequently. Furthermore, the per-cell rate of this initial phage adsorption is assumed to not decline despite bacteria being present within these clumps, i.e., at least for now *k* is assumed to remain constant across cells within clumps. In this case, then we can present as an alternative equation the following,

$$\frac{dP}{dt}=nBPkN-PX.$$ (3)

In words, the global rate of change of phage densities (*dP*/*dt*) is equal to *n* bursts (*B*) for every initial phage adsorption of a bacterium (*PkN*) that is part of a clump made up of *n* bacteria, subtracted by an adsorption-independent rate of inactivation of phage virions (*PX*). To better illustrate what is going on, we can thus separate the production aspect of the equation (*nB*) from the adsorption aspect (*PkN*),

$$\frac{dP}{dt}=\left[nB\right]\left[PkN\right]-PX.$$ (4)

Ignoring these brackets and manipulating this equation equivalently to as above, then

$$N_{\text{T}}=X/nBk,$$ (5)

which in turn implies that *N*_T_ ∝ 1/*n*. That is, the proliferation threshold is inversely proportional to the number of cells found per clump. Since fractions become smaller in magnitude as denominators increase, *N*_T_ for bacterial clumps, i.e., of size *n* > 1, therefore should be smaller than *N*_T_ for isolated, individual bacteria (*n* = 1).

The implication is that the overall density of bacteria necessary to support the persistence of a phage population is smaller the more phage infections which an adsorbed entity can support in total, clump versus isolated bacterium. This occurs because, though passive adsorptions will occur at the same rate with or without clumping (i.e., as so-far assumed), active adsorptions will occur at a much higher rate within phage-infected clumps, resulting in relative rates of virion inactivation which are lower—implicitly here the virion inactivation rate within clumps is assumed to be equal to zero. That is, we are assuming that newly infection-released phages are much more likely to succeed in infecting a bacterium rather than becoming inactivated as virions if the to-be-infected bacterium is found within the same bacterial clump which initially produced and then released a phage. Thus, the total number of bacteria required to support the persistence of a phage population globally should, at least tentatively, be smaller with clumping than without (answer c, “greater potential”, Section 3.1.3).

By way of example, given *n* = 1 (isolated individual bacteria), a rate of phage inactivation of 0.01/min (1%), a burst size of 100, and the value of *k* of 2.5 × 10^−9^ ml/min, the latter as from Stent [34], then *N*_T_ = 4 × 10^4^ bacteria/ml (which, reiterating from Section 3.2.1., should be seen as far too small to globally support the production of inundative phage densities, though in fact this represents the proliferation threshold bacterial density). If *n* is set equal to 10, then we have *N*_T_ = 4 × 10^3^, which is ten-fold lower and assumes that both *k* and *X* otherwise remain constant. We also are ignoring that it will take more time for phages to propagate through a clump of bacteria than it takes to lyse a single bacterium, e.g., at least twice as long as representing at least two phage latent periods. Note, though, that all of these proliferation threshold equations as presented are independent of delays between phage adsorption and burst, and therefore the duration of these post clump-adsorption processes will not otherwise be considered. Notwithstanding this detail of timing, proliferation thresholds given bacteria presence within clonal clumps should be lower, at least as considered so far (again, answer c, Section 3.1.3), versus cultures consisting of isolated, individual bacteria. Can this claim, however, hold up to closer scrutiny?

#### 3.2.3. Globally active versus locally active treatments

Contrary to assumptions made immediately above, the burst size supported by every cell making up a clonal bacterial clump in fact may not be equivalent, with some bacteria within clumps being physiologically unable to support otherwise normal phage bursts. Also, phage likelihood of encountering bacteria may be reduced given the shading of bacteria by other, closely associated bacteria, that is, reductions in the number of bacteria with which phages may easily collide given a piling of bacteria upon each other within clumps. Per unit time this may have the effect of reducing numbers of bursts by reducing rates of phage collision with bacterial clumps. Furthermore, if the assumption of only a single passive adsorption per clump is relaxed, then the number of phage bursts which can occur per passive adsorption, i.e., *n*, in fact will be smaller, that is, fewer new phages per passive adsorption. It is conceivable as well that clump-associated mechanisms could increase rates of virion inactivation, most notably phage adsorption of already phage-infected bacteria. Smaller burst sizes, slower adsorption, fewer bursts, or greater rates of phage inactivation given clumping [8],[44] have the effect of lowering the number of phages which are produced or which survive per phage-adsorbed clump.

Given these various issues, it is conceivable that *n* in terms of sustaining phage populations across environments, that is, rather than simply being a measure of cell number per clump, could very well be equal to or at least approach a value of 1. That is, clumping could have little or no impact on bacterial proliferation threshold densities and therefore on the potential for phages to display active treatment, closer to answer (a), “no difference”, rather than answer (c), “greater potential” (Section 3.1.3.). This conclusion, however, is not necessarily true at all spatial scales. That is, active treatment by definition is phage therapy that requires *in situ* phage population growth to produce sufficient phage numbers to, ultimately, inundate bacteria. There is a distinction, though, between sufficient numbers across environments (i.e., globally) versus sufficient numbers locally, that is, at smaller distances such as in association with isolated bacterial clumps as can be viewed on sub-millimeter spatial scales, i.e., locally.

In the previous section (3.2.2.) a calculation was made for a proliferation threshold of 4 × 10^4^ bacteria/ml given a phage burst size of 100, *k* = 2.5 × 10^−9^ ml/min, and 1% per min rate of free virion decay. In Section 3.1.1., by contrast, bacterial densities at least in the range of 10^6^/ml were suggested as being necessary, given burst sizes of 100—at that point, ignoring virion decay—to reach what could be minimal levels inundative phage densities. Given clumping, bacterial densities as found extremely locally, however, can massively surpass both of these numbers, e.g., with bacterial densities easily exceeding 10^8^ bacteria/ml [8]. Consequently, even given insufficient bacteria to support or even sustain phage population densities across environments, active phage treatment could still be possible given clumping since local bacterial densities in association with clumps can be very high. Such active treatment, however, would be seen only on local (<<1 millimeter) rather than on more global scales, that is, answer (c), “greater potential” (Section 3.1.3); i.e., effecting locally active treatment but not or at least less so effecting globally active treatment).

Thus, we have rejected the idea that clumping will necessarily substantially contribute to a lowering of those threshold bacterial densities necessary, at a minimum, to support globally active treatment, but have not rejected the idea that clumping nevertheless could support locally active treatment. Phages nonetheless need to reach clumps in order to initiate their localized population growth. This implies that sufficient phage numbers still must be supplied to result in timely initial, i.e., passive phage adsorptions. Such adsorptions should be more likely for individual bacterial clumps given their larger target sizes versus individual bacterial cells, and these larger target sizes should be seen even with bacteria shading other bacteria from phage adsorption.

In the next sections we consider how bacteria presence within such clumps might affect what phage numbers must be applied via dosing to result in a global reaching of inundative phage densities, especially when densities of target bacteria nonetheless are insufficient across environments to support active treatments more globally. That is, this is towards achieving active treatment at local scales even if active treatment, as more traditionally defined (Table 2), cannot be achieved over global scales, or indeed how clumping could serve to accelerate rates of bacterial eradiation. Generally the idea is developed that inundative densities may be lower given clumping versus without, though only if phages are competent to replicate under treatment conditions.

### 3.3. Viral eradication threshold (minimum inhibitory concentration)

A key aspect of appreciating the pharmacology of antibacterial agents is the concept of minimum inhibitory concentration (MIC). This, as the name implies, is the lowest antibacterial concentration which has the effect of preventing bacterial population growth. Thus, upon exposure to an MIC of, for example, an antibiotic, a bacterial population will not increase in size, but at lower dosages it will. With phages the determination of an MIC, unfortunately, is more difficult than it is for small-chemical antibiotics owing to a combination of single-hit killing kinetics by phages and the ability of phages to replicate, the latter which results in positive changes in phage concentrations. The complication of phage replication, however, is not seen if employing phages which are bactericidal but not able to productively infect target bacteria [45],[46]. MIC determination for bacteriophages is not just difficult to accomplish, it also does not have equivalent meaning as MIC for antibiotics since a key reason for exploring MICs for antibiotics is an antibiotic's potential for toxicity at higher concentrations, hence a utility to defining *minimum* inhibitory concentration. Toxicity, however, is thought to be less of a concern especially for well-purified phages [47],[48]. For these various reasons, MIC determinations are just not especially useful for calculating dosing for phage therapy [15].

#### 3.3.1. MIC alternative

There exists an alternative approach to obtaining MIC-like information for phages. This is the calculation of what is known as a viral inundation threshold [12], a.k.a., a minimum inundatory dose [11]. An extension of the idea of inundation threshold is addressed here, specifically that of an “eradiation threshold”, which unlike inundation thresholds explicitly takes into account some degree of phage population growth, i.e., as in association with bacterial clumps (locally active treatment). Note that “thresholds” in this case are measured in viral densities, that is, rather than those of bacteria, while both “inundation” and “eradication” are by phages and of bacteria. In addition, the concept of “inundation threshold” is different from that of “inundative density” (Section 3.1.), where the former, like threshold bacterial densities (Section 3.2.), represents a minimum rather than adequate or desirable density whereas an inundative density, as defined here, is that phage density that is both adequate for reducing bacterial densities (versus inadequate) and desirable in that it is sufficiently large to succeed in reducing bacterial densities in a timely manner.

An inundation threshold is that phage density which is sufficient to prevent a bacterial population from growing, with this inhibition accomplished specifically by phages killing bacteria. Phage densities which are lower than the inundation threshold will allow the bacterial population to continue to grow in size whereas phage densities which are greater than the inundation threshold will result in a killing off of the bacterial population. Unfortunately, this idea of killing off a bacterial population is itself fraught with complication since one must also take into account issues of time frames over which this bacterial killing takes place (re: inundative densities) as well as the degree to which one desires bacterial populations to be eliminated, e.g., to 10^−2^ (i.e., 1% survival), 10^−4^ (i.e., 0.01% survival), etc. With inundation thresholds, however, these issues actually are resolved or, at least, avoided. Less easily resolved, however, are assumptions that inundation occurs as a consequence of phage dosing (passive adsorptions only) rather than as a result of *in situ* phage population growth (active adsorptions as well). If we consider only the former circumstance, that is, where phage densities are a function solely of phage dosing, then inundation therapy is equivalent to passive treatment. Indeed, Payne and colleagues [11],[12],[13] explicitly equate these concepts.

If one strictly considers an inundation threshold in which phages are not allowed to replicate, then at best there will be no difference between phage treatment of isolated bacteria and phage treatment of clumped bacteria. Indeed, to the extent that clumped bacteria can shade each other from phage attack (Section 3.2.3.), then inundation should require greater time spans when bacteria are found in clumps versus bacteria which are not clumped. If phages are bactericidal but not bacteriolytic, then phages may not even be able to reach shaded bacteria [8],[49]. Thus, for clumped bacteria one cannot just take into account phage killing ability but must include as well phage infection ability. As a consequence of these considerations, the concept of inundation threshold is unsatisfactorily applicable to phage treatment of clumped bacteria. I therefore propose, as a more general term, eradication threshold. This, as defined here, is that concentration of phages which are able to exactly offset rates of bacterial population growth given some degree of especially only localized phage replication (i.e., as over <<1 millimeter scales), that is, as within bacterial clumps.

For the sake of simplicity in both calculations and their interpretation, an assumption is made that while phage replication has the result of increasing phage densities locally, i.e., within clumps, there is no appreciable increase in phage densities more globally, such as between clumps. Low rates of environmental mixing, such that newly produced virions have difficulty diffusing between bacterial clumps, will also have the effect of supporting an assumption of reduced individual-phage global impact even if phage densities are able to increase in number in the course of treatment of environments. An assumption is being made, that is, that initial adsorptions of bacterial clumps consist solely of passive adsorptions. In terms of treatment of solid or semi-solid foods (Appendix), this assumption is consistent with more effective phage diffusion to bacteria sooner after application, i.e., before drying or absorption of carrier fluids takes place, thereby reducing virion mobility over time.

#### 3.3.2. Estimating inundation thresholds

For an inundation threshold, threshold refers to that minimum density of phages which are capable of preventing bacterial population growth. If bacteria are replicating, and that replication is considered to be negatively impacted only by phage adsorption, then this threshold is seen when the density of phages is such that for every new bacterium produced via bacterial replication, one bacterium is lost as a consequence of phage adsorption. The calculation provided by Payne and colleagues [11],[12] to represent this balance is extremely simple,

$$P_{\text{I}}=\frac{µ}{k},$$ (6)

where *µ* is the instantaneous rate of bacterial growth known as the Malthusian parameter and *k* is the phage adsorption rate constant. *P*_I_, in turn, is the phage density that is equal to the inundation threshold. Intuitively, when

$$P_{\text{I}}k=µ,$$ (7)

then individual bacteria are being adsorbed by phages at a rate (left side of equation) that is equal to the rate that bacteria are dividing (*µ*).

As MIC is defined as the minimum antibacterial concentration capable of preventing bacterial population growth, in a sense *P*_I_ is MIC, though with the caveat that the calculation is dependent on phage population densities not changing over time. In addition, and as noted, bacterial populations are not actually being reduced in density in this scenario but, rather and by definition, are remaining at the same population density. Thus, at best, this calculation is a useful way of ascertaining what phage densities may be grossly inadequate to achieve bacteria-killing efficacy based on passive treatments.

Though inadequate as a measure of what phage densities are necessary to eradicate target bacteria, especially in a timely manner, this calculation nevertheless does indeed represent a threshold that falls between, on the one hand, sufficient phage densities to reduce bacterial numbers over time—even though that time can be extremely long depending on how close phage densities are to this threshold—and, on the other hand, insufficient phage densities to prevent net bacterial population growth. Again, keep in mind that phage population densities are assumed in this model to not change over time. Consequently, I employ a similar but nonetheless alternative threshold—one which does allow some phage replication—in order to extend the above model to bacteria which are clumped and thereby are potentially subject to locally active treatment. This I describe as a bacterial-“eradication” threshold.

#### 3.3.3. Considering eradication thresholds

Calculation of an eradication threshold will not be explicitly attempted. Instead, it is its magnitude relative to the inundation threshold that will be considered. Related issues, however, will be addressed in subsequent sections.

Here a subscript of “I” pertains to Inundation thresholds, that is, as concerning individual, isolated bacteria, whereas a subscript of “E” refers to Eradication thresholds, as pertaining especially to bacterial clumps. Three assumptions are made. The first is that the rate of bacterial population growth within clumps is either equal to or lower than that of isolated bacterial populations existing within the same environment, thus, *µ*_E ≤_ *µ*_I_ (meaning that bacterial growth rates when considering eradication thresholds of bacterial clumps are less than or equal to bacterial growth rates when considering inundation thresholds of individual bacterial cells). Bacterial growth rates across biofilms in particular can vary given spatially heterogeneous access by constituent cells to nutrients or oxygen [50], that is, across individual clumps, as potentially can result in lower average rates of bacterial growth.

The second assumption is that the target size of bacterial clumps is greater overall than that of individual bacteria [33]. Note, however, that this statement does not directly address the question of whether the target size of individual cells making up clumps differs from that of cells not found in clumps, i.e., the issue of bacteria shading each other (Section 3.2.3). The overall target size of bacterial clumps thus is likely smaller than the sum of bacteria making up a clump, here indeed as due to shading of bacteria by bacteria (Section 3.2.3.). Nevertheless, the collective target size of multiple cells making up a single clump still should be larger than that of a single cell. As the phage adsorption rate constant or, more precisely, the collision kernel increases proportionally with target size, then we have an expectation not just of *k*_E ≥_ *k*_I_ but indeed of *k*_E_ > *k*_I_, that is, the adsorption rate constant to an entire bacterial clump (*k*_E_) should generally be greater than the adsorption rate constant for individual, isolated bacteria (*k*_I_) (Sections 3.5.2. and 3.5.3.).

The third assumption is that once a clump has been adsorbed by a phage, then the clump is completely eradicated in the course of subsequent, extremely localized (<<1 millimeter) phage population growth (Sections 3.1.3. and 3.2.3.). For simplicity, envisage the treatment of poorly mixed semi-solid foods with phages where, due to relatively low bacterial loads at the start of treatment, phage densities will tend to increase extremely locally within the vicinity of phage infections but not appreciably over the entirety of the treated food. These local increases in phage densities consequently can result in active treatment only over extremely local scales, i.e., as coinciding with an isolated clump of bacteria (<<1 millimeter).

If we make these assumptions, then we can reformulate *P*_I_ as *P*_E_, indicating how the different parameters may be modified in going from one threshold to the other. Thus,

$$P_{\text{E}}=\frac{\leq µ}{\geq k}.$$ (8)

As decreases in numerators or increases in denominators both result in decreases in the size of a fraction, we can conclude that *P*_E_ ≤ *P*_I_, or indeed *P*_E_ < *P*_I_ assuming either *k*_E_ > *k*_I_ or *µ*_E_ < *µ*_I_. Since *P* is phage density, this suggests that the number of phages required to control the replication of populations of clumped bacteria would be lower than as required were bacteria not clumped. It likely follows that, given constant phage densities, then bacterial eradication will occur more rapidly given bacterial existence within clonal clumps versus as isolated cells. The next sections (3.4. and 3.5.) explore this latter prediction.

### 3.4. Timing of phage-mediated eradication of bacterial clumps

Considered in this section are a number of ways of describing the rate with which phage populations can impact bacterial populations, again distinguishing the impact on bacterial clumps from that on isolated, individual bacteria. These approaches include prediction of the bacterial mean free time, prediction of bacterial half lives, and prediction of a bacterial decimal reduction time. Though not entirely equivalent, nonetheless all of these concepts are defined similarly. So too, in the following section (3.5.), is time until bacterial eradication addressed, though that calculation requires additional considerations.

#### 3.4.1. Mean free time

Given a constant phage density, then bacterial populations will decline in size exponentially as a consequence of phage adsorption. One way to indicate these rates of decline is in terms of a mean free time (*t*_m_), as equivalent to the mean free path from physics. This is the average duration that a bacterium will remain phage-free in the face of phage adsorption. For individual bacteria this mean free time is simply,

$$t_{\text{m}}=\frac{1}{kP},$$ (9)

where *k* is the phage adsorption rate constant and *P* is phage density. Note the distinction between bacterial mean free time and that of phage mean free time, where the latter (=1/*kN*) instead is the average time of phage persistence while in association with a given density of phage-susceptible bacteria [51],[52].

If we set *n* again to the number of bacteria making up a bacterial clump, or at least the increased target size of a bacterial clump relative to a single bacterial cell (Section 3.2.3.), then the clump mean free time can be approximated as,

$$t_{\text{m}}=\frac{1}{nkP}.$$ (10)

Thus, like the proliferation threshold of bacterial densities for phage population growth (*N*_T_, Section 3.2.2.), we can estimate that *t*_m_ ∝ 1/*n*. That is, the larger the bacterial clump—or faster phage adsorption or greater phage densities—then the shorter the bacterial clump's mean free time. We therefore would predict the following:

$$\frac{1}{kP}>\frac{1}{nkP}.$$ (11)

That is, individual, isolated bacteria should remain unadsorbed over a longer time, for a given phage density, than individual bacterial clumps.

Another way of stating this is that physically linked together or not, inevitably there will be a higher likelihood that at least one bacterium of a group of bacteria will become phage adsorbed versus a single bacterium (Section 3.3.3.), resulting in a shorter mean phage-free time for a group of bacteria versus a solitary bacterium. Alternatively, the mean free time could remain the same but at lower phage densities. The implication is that inundative phage densities could be reached at lower *in situ* phage densities given bacterial clumping versus without.

#### 3.4.2. Half life of a bacterial clump

“Half” describes the point at which 50% of all targets for “passive” phage adsorption—individual, isolated cells or instead bacterial clumps—have become phage adsorbed. Because these entities will display an exponential decline given exposure to phages, this adsorption of half will come sooner than the average, or “mean” length of time until phage adsorption. This distinction occurs because one must take into account an infinitely trailing tail of lack of complete adsorption when calculating a mean free time. That is, exponential declines, at least mathematically, never reach zero. Thus, the mean free time of a bacterium or clump of bacteria in the face of phage adsorption is not identical to the pre-adsorption half life of a population of bacteria, or of bacterial clumps.

An additional distinction is that between “life” and “free”. At a minimum, “life” can be equated with not inactivated or not decayed, or not eradicated, whereas “free” refers to not adsorbed (or, more generally, not collided with). Half life thus refers to a point at which half of a population of isolated cells or instead of bacterial clumps have not yet been eradicated by phages. Eradication of a bacterial clump occurs in the course of localized phage population growth, that is, as in association with the clump (locally active treatment). Such eradication can be viewed as equivalent to the loss of colony-forming units (CFUs) following phage adsorption, with individual clumps serving as such units and CFU eradication equivalent to a loss of clump “life”. Such clump eradication is less dependent on overall phage productivity than is global generation of inundative phage population densities (Section 3.2.2.). Particularly, there should be less dependence on phages displaying relatively large burst sizes so as to infect neighboring bacteria within a clump versus populating an entire environment with inundative densities of phages. The magnitude of *n*, even though we have switched from considering primarily phage adsorption (mean free time) to phage-mediated eradication of bacteria or clumps (half life) should thus remain a function especially of target sizes rather than also substantially of per-cell phage productivity (i.e., burst sizes).

Notwithstanding these distinctions, the bacterium or bacterial clump mean free time given phage adsorption still serves as an approximation of the bacterium or bacterial clump half life given phage-induced mortality (*t*_½_). Thus,

$$t_{\text{½}}=\frac{0.69}{nkP}\approx \frac{1}{nkP}=t_{\text{m}},$$ (12)

where 0.69 = –ln(0.5). The left-side of the equation, as indicated, rounds up to 1, and thus *t*_½_ can be rounded up to the mean free time. In any case, note equivalently that *t*_½_ ∝ 1/*n*. Where specifically the –ln(0.5) comes from is explained in the following subsection (3.4.3.).

#### 3.4.3. Decimal reduction time

An alternative perspective to that of bacterial mean free time or half life, in terms of rates of reduction in bacterial densities that result from phage adsorption, is decimal reduction time (*D*). This is how long it takes for a phage population to reduce the density of a bacterial population ten-fold, i.e., by 90%. The half-life measure, by contrast, is the time (*t*_½_ = *t*_0.5_) that it takes to reduce bacterial densities by 50%. Setting the equivalent *t*_0.1_ to *D* for *D*ecimal reduction time, we have simply [15],

$$D=\frac{2.3}{nkP},$$ (13)

where 2.3 = –ln(0.1). Thus, as with bacterial half life as well as bacterial proliferation threshold density and mean free time, *D* ∝ 1/*n*.

For both decimal reduction time and bacterial half life, note that their derivation is from this equation,

$$N_{t}=\;N_{0}e^{-kPt},$$ (14)

where *N*_t_ and *N*_0_ are bacterial density following *t* min of phage exposure and bacterial density prior to phage exposure, respectively. *N*_t_/*N*_0_ thus is the fraction of bacteria remaining following time = *t* of phage adsorption, e.g., 50% (0.5) or 10% (0.1). Rearranging, we have,

t=−ln(Nt/N0)kP or t=−ln(Nt/N0)nkP, (15)

where *t*, for time, thus is equivalent to *D* or *t*_½_, etc., depending on the ratio of *N_t_* to *N*_0_.

### 3.5. Time until bacterial eradication

The time it takes to bring a bacterial culture under control is a function, in part, of how “bring a bacterial culture under control” is defined. In terms of bacterial densities, a simple means of doing this is to define the dynamics of bacterial death, as above, as an exponential function. Thus, for example, less control is observed given survival of 10^0^ bacteria/ml rather than 10^−2^ bacteria/ml or 10^−4^ bacteria/ml, etc. The issue considered in this section is whether bacterial existence within clumps will contribute to a bacterial population being brought under control sooner versus given bacteria existing instead as isolated cells. As above, an assumption is made that a single phage adsorption to a single clonal bacterial clump is sufficient to result in the death of all phage-sensitive bacteria making up that clump (see Section 4. for justification). These calculations, though similar to those presented in Section 3.4., nevertheless can be more complex.

#### 3.5.1. Preliminaries

The first step in calculating time until bacterial eradication is to extend the calculations presented in Section 3.4. The calculation itself is equivalent to that discussed under the heading of decimal reduction time (Section 3.4.3.) since bacterial survival again is indicated by the ratio of *N_t_* to *N*_0_. Thus, if we define a time until bacterial eradication, *t*_E_, then this simply is equal, as above, to,

tE=−ln(Nt/N0)kP or tE=−ln(Nt/N0)nkP, (16)

where the former equation is without clumping and the latter with.

If we define bacterial eradication as survival at a level of 10^0^ bacteria/ml (=*N_t_*), but start with 10^3^ bacteria/ml (=*N*_0_), for example, then –ln(*N_t_*/*N*_0_) = –ln(10^−3^) = ln(10^3^) = 6.9. Thus, with clumping,

$$t_{\text{E}}=\frac{6.9}{nkP}.$$ (17)

Not surprisingly, at this point in the motivation of this calculation, we find again that time until bacterial eradication, like bacterial mean free time, bacterial half life, and decimal reduction time, is inversely proportional to *n.* For *n* = 10 cells, *k* = 2.5 × 10^−9^ ml/min, and *P* = 10^7^ phages/ml, *t*_E_ thus works out, for example, to 27.6 min with versus 276 min without clumping, starting with 10^3^ bacteria/ml and ending up with a single bacterium/ml (=10^0^ bacteria/ml). The ten-fold difference is entirely a function, here, of an increased clump target size relative to that of individual bacteria, again assuming that a single phage adsorption per clump results in complete loss of the entire clump. We return to this issue of target size in the following section.

Though consistent with as seen in the previous section, this time-until-bacterial-eradication result is only preliminary since, unlike with those previous calculations (Sections 3.4.2. and 3.4.3.), rather than describing a relative decrease (e.g., 50% or 90%), bacterial eradication represents an absolute reduction, i.e., to a specific density of remaining bacteria. In other words, unlike half-lives, decimal reductions, and also mean free time, the fractional decrease changes as a function of starting bacterial density, such that it is larger if you start with more bacteria and smaller if you start with fewer. As a consequence of this change, it becomes important to take into account absolute reductions in the number of bacteria and how bacterial clumping can impact that number. This impact is seen particularly in what constitutes the end-point of bacterial eradication, that is, at what point in terms of numbers of bacterial clumps eradicated can sufficient bacterial eradication be declared?

#### 3.5.2. End-point considerations

As indicated immediately above, we are defining the point of bacterial eradication as some resulting bacterial density such as, for example, one cell per ml. Because we are assuming that a single phage adsorption to a bacterial clump results in complete eradication of that clump (see the introduction to Section 4. for further discussion of this assumption), the result is that *n*-fold fewer primary phage adsorptions need occur, assuming for the presented comparison the occurrence of no more than one passive adsorption per adsorbed clump, or per adsorbed isolated bacterium. Thus, if we start with 10^3^ bacteria (*N*_0_) and want to reduce this number to 10^0^ bacteria (*N_t_*), then this will take at least 10^3^ – 10^0^ ≈ 10^3^ passive adsorptions without clumping, but given a clump size of *n* = 10 cells, then a minimum of ∼10^2^ passive adsorptions, plus 9 active adsorptions for every clump adsorbed. Furthermore, the ratio of starting target density (*N*_0_/*n*) to desired ending bacterial density (*N_t_*) is equal to *N*_0_/*nN_t_*.

At this point we have to directly confront the idea that *n* in terms of the target size of clumps and *n* in terms of the number of bacteria making up individual clumps may not be identical. Specifically, due to bacteria shading each other (Section 3.1.3.), *n* as indicating target size probably is smaller than *n* as indicating cell number. It is beyond the scope of this study to consider exactly what this difference might entail (though see Section 3.5.3. for initial discussion), but to address it we can consider an *n* for target size, or *n*_ts_, versus an *n* for cell number, or *n*_cn_. Thus distinguished, we can incorporate an expression, *n*_cn_*N_t_*, as well as *n*_ts_ into the above solution for *t*_E_,

$$t_{\text{E}}=-\frac{\text{ln}\left(n_{\text{cn}}N_{t}/N_{0}\right)}{n_{\text{ts}}kP}=\frac{\text{ln}\left(N_{0}/\left(n_{\text{cn}}N_{t}\right)\right)}{n_{\text{ts}}kP}.$$ (18)

Repeating the previous example calculation (Section 3.5.1.) for *N_t_* to *N*_0_ = 10^−3^ as well as *n*_cn_ = 10 and *P* = 10^7^ phages/ml, then we have,

$$t_{\text{E}}=\frac{4.6}{n_{\text{ts}}kP}.$$ (19)

Further, we can argue that target size might be reduced by, for example, half. Thus, *n*_ts_ would be equal to 5 instead of 10, and thereby,

$$t_{\text{E}}=\frac{4.6}{5kP}=36.8\approx \frac{1}{kP}\text{ versus }t_{\text{E}}=\frac{6.9}{1kP}=276,$$ (20)

the latter assuming no clumping. In this case, the result would be a bit less than a ten-fold shorter time until bacterial eradication [6.9/(4.6/5) = 7.5 = 276/36.8] given bacterial clumps of 10 cells each versus without clumping. It must be stressed, however, that this calculation is at best only an approximation.

#### 3.5.3. Solutions

From the calculation presented in Section 3.5.1., we can conclude that clumping can have an impact on time until bacterial eradication by reducing that time, that is, by having a negative impact on *t*_E_. This reduction is a consequence of clumps displaying larger target sizes than individual bacteria (Section 3.3.3.). Thus, phages find individual clumps faster in terms of passive adsorptions. Active adsorptions, resulting in clump eradication, are then assumed to subsequently occur in the course of localized phage population growth. In principle, passive adsorptions can occur faster than active adsorptions—given the latter's requirement for at least one phage latent period prior to the start of such adsorptions—but this is certainly the case only given dosing with sufficient phage densities that all bacteria are rapidly overwhelmed with passively adsorbing phages. Use of extremely high phage densities is not the emphasis here, however, but rather how bacterial presence within clonal clumps may allow for enhancements in antibacterial efficacies without overly emphasizing passive treatment strategies to reduce bacterial numbers, i.e., without resorting to extremely high densities of applied phages (Section 3.1.2.). Indeed, the higher the phage densities that are applied, then the more similar should be delays until bacterial eradication with versus without clumping as multiple phage adsorptions of individual bacterial clumps would have the effect of reducing the magnitude of *n*, that is, the number of bacteria killed per passive adsorption (Sections 3.2.2. and 3.2.3.).

A second issue, considered in the previous section (3.5.2.), is the impact of clumping on what constitutes the endpoint of bacterial eradication, that is, as impacting *N_t_* via *n*_cn_. This effect should also reduce the time until bacterial eradication (negative impact on *t*_E_), though as a function of the natural log of the number of cells making up clumps. We thus have an expectation not only of faster passive adsorption to clumps versus individual bacteria (previous paragraph) but also a need for fewer passive adsorptions, as active adsorptions take over to complete the process of bacterial eradication.

The third issue again concerns target sizes, but is a complication on that as depicted in Section 3.5.1. Though larger than individual bacteria, clump target sizes still are likely smaller than the sum of the target sizes of constituent bacteria, i.e., as due to bacteria shading other bacteria (Sections 3.3.3. and 3.5.2.). Such less than maximal increases in the target size of clumps as a function of numbers of constituent cells should result in smaller negative impacts of target sizes on *t*_E_ than as suggested in Section 3.5.1. This is considered in the last set of calculations presented in Section 3.5.2 as a reduction in *n*_ts_ relative to *n*_cn_. Clump target sizes and thus *n*_ts_ actually could vary as a root function of cell number, versus simply assuming that target sizes are cut in half, as was done in Section 3.5.2. At an extreme, this would be a third root, that is, considering clumps as idealized spheres which increase in radius as a function of the cube root of volume, where volume increases as a direct function of cell number (i.e., sphere radius is proportional to the third root of sphere volume). Thus, at a minimum, *n*_ts_ ∝ *n*_cn_^1/3^, and this is rather than simply *n*_ts_ ∝ *n*_cn_, where the latter would give rise to a larger negative impact by *n*_cn_ on *t*_E_ (*n*_cn_ as cell number per clump), while the former would give rise instead to a cube-root (i.e., smaller) negative impact by *n*_cn_ on *t*_E_.

Table 3 presents additional solutions to the equation derived in Section 3.5.2. for various values of *P* (=phage density) and *n* (equals cell number per clump), setting *k* equal to 2.5 × 10^−9^ ml/min, and defining *n*_ts_ as the cube root of *n*_cn_. Note that with 100 bacteria/clump and 10^7^ phages/ml, the time until bacterial eradication is 20 min (2.0E+01), but 86 min (8.6E+01) given a clump size of 10, and 280 min (2.8E+02) for bacteria not found in clumps. The latter, however, is reduced to 28 min given instead application of a phage density of 10^8^/ml, and indeed to 8.6 min (8.6E+00) given a clump size of 10 and 10^8^ phages/ml, but is 860 min (8.6E+02) with *n* = 10 and *P* = 10^6^, the latter versus 2800 min (2.8E+03) without clumping. It is not possible, however, for 10^4^ bacteria/ml to support phage population growth to 10^8^/ml across an environment (globally active treatment), unless phage burst sizes are equal to roughly 10,000. By contrast, there can be substantial differences with versus without clumping in the potential for bacteria to support phage population growth to inundative densities, particularly over very small, clump-sized spatial scales (Section 3.2.3.). Indeed, in very general terms, it typically is easier to have a larger impact on multiple targets if they are found in close proximity versus instead dispersed across environments, and especially so given a potential by antagonists for “explosive” local propagation. Thus, with clumping, phage access to bacterial targets (i.e., bacterial clumps versus individual, isolated bacteria) is predicted to occur over shorter time scales, or instead may be achieved over similar time scales using fewer phages, though with the caveat that for active treatment to function, then phages once they have reached target bacteria *in situ* must retain a potential to replicate.

**Table 3.** Time (in min) until Bacterial Eradication, Going from 10^3^ to 10^0^ bacteria/ml.*

*n* ↓ *P* →	10^2^	10^3^	10^4^	10^5^	10^6^	10^7^	10^8^	10^9^	10^10^
1	2.8E+07	2.8E+06	2.8E+05	2.8E+04	2.8E+03	2.8E+02	2.8E+01	2.8E+00	2.8E–01
10	8.6E+06	8.6E+05	8.6E+04	8.6E+03	8.6E+02	8.6E+01	8.6E+00	8.6E–01	8.6E–02
100	2.0E+06	2.0E+05	2.0E+04	2.0E+03	2.0E+02	2.0E+01	2.0E+00	2.0E–01	2.0E–02
500	3.5E+05	3.5E+04	3.5E+03	3.5E+02	3.5E+01	3.5E+00	3.5E–01	3.5E–02	3.5E–03

* *P* is in units of constant phage virions/ml while *n* is in units of bacterial cells/clump. With E notation, for example, 8.6E+01 = 8.6 × 10^1^ = 86 min. Time until eradication, *t*_E_, is defined as in Section 3.5.2., with *n*_ts_ set equal to the cube root of *n*_cn_ = *n*. Eradication is as would be measured in terms of bacteria plating without disrupting colony-forming units, i.e., where the presence of one phage per clump, with a clump serving as a potential CFU, is sufficient to prevent subsequent colony formation. Keep in mind, however, that time going from the point of passive phage adsorption to a bacterial clump and complete eradication of the resulting phage-adsorbed clump (as mediated through active phage adsorptions) has not been taken into account in the table.

## 4. Discussion

For phage-mediated biocontrol to be successful, then target bacteria must be both reached by phages and susceptible to those phages during the treatment process. If bacteria cannot be reached by phages then some form of environmental modification or improved phage delivery must be undertaken. If bacteria are not susceptible, then new, more effective phage types must be obtained. Phages in addition must reach target bacteria in sufficient quantities to ultimately result in some approximation of bacterial eradication. Phage infections in combination with phage-induced bacterial lysis may to some degree also “clear the way” for further productive or at least bactericidal or bacteriolytic phage infections, a phenomenon I've described elsewhere as an “active penetration” of phages into bacterial biofilms [9],[49]. The idea of phage bursts occurring in the immediate vicinity of other bacteria within clonal bacterial clumps, thereby resulting in locally higher phage densities, I've described elsewhere as a bacterial “spatial vulnerability” [33]. The key point here, though, is that with or without active penetration or spatial vulnerability, successful phage-mediated biocontrol simply cannot occur without a combination of sufficient phage access to target bacteria, sufficient susceptibility of bacteria to phages given that access, sufficient numbers of bacteria-accessing phages, and also sufficient time frames over which phage access to bacteria can occur.

Given this logic, if bacterial clumps are not mostly or fully both reachable by and susceptible to the phages used for their biocontrol, then eradication of those bacteria using these phages simply cannot occur. This furthermore would be true whether involving passive treatment, only locally active treatment, or instead globally active treatment. In other words, here we are not asking the question of whether bacterial clumps are or are not phage susceptible but instead indicating, especially during phage-mediated biocontrol of food-contaminating bacteria, that if treatment is going to be successful then at a minimum a sufficient fraction of targeted bacteria in fact must be both phage reachable and phage susceptible under the conditions employed. It nevertheless would be given conditions in which bacteria have had some potential to replicate *in situ* prior to phage application that locally active treatment/spatial vulnerability may come into play, but only if the phages used are also sufficiently capable of replicating, *in situ*, in the presence of these bacteria, plus are given sufficient time to do this replicating. Microbiome editing using phages, especially of minority populations of problematic bacteria, may similarly possess an enhanced potential to succeed given an opportunity by phages to display a locally active treatment. This Discussion thus focusses especially on exploring the circumstances in which achieving a locally active treatment may be useful, especially within the context of phage treatment of foods but also of microbiomes more generally. Further discussion of phage-mediated biocontrol of bacteria within foods can be found in the Appendix.

### 4.1. Minimum effective phage concentrations

Certainly it is possible to apply overwhelming concentrations of an antibacterial agent to targeted bacteria. This would be amounts which are well in excess of minimum inhibitory concentrations (MICs). There will be issues with employing very high concentrations, however, issues which are associated with either the economics of treatment or instead the potential for antibacterial agents to damage treated systems. MICs thus can serve as a dosing guide, with *in situ* antibacterial densities ideally exceeding MICs, rather than being lower than MICs, but not exceeding MICs to so great an extent as to, in various ways, become impractical. As noted, MIC determination for phages can be problematic for a number of reasons (Section 3.3.). Nevertheless, some minimum concentration of phages must exist which is capable of getting a given antibacterial job done—assuming that efficacy can be accomplished at all—and this can be viewed as a minimum effective concentration, or MEC.

For phage-mediated biocontrol, an MEC is a sufficient *in situ* density of phages following dosing to allow, ultimately, for phage adsorption to some approximation of all targeted bacteria over a reasonably short span of time (Section 3.5.). What constitutes an MEC is going to be situation specific, though nevertheless an MEC is that phage concentration which is capable of either serving on its own or instead giving rise to an inundative phage density (Table 2). Inundative phage densities by contrast are those concentrations which are sufficient on their own to eradicate bacteria in a timely manner (Section 3.1). A phage titer of 10^8^ phages thus may represent an inundative phage density. An MEC for the same system, however, might consist of, e.g., an *in situ* phage density of 10^6^ or 10^7^ phages per ml, with the difference between MEC and resulting inundative phage densities both attributable to and requiring active phage replication, *in situ*.

With active treatment, *in situ* phage population growth thus is required for MECs to give rise to inundative phage densities, whereas with passive treatment MECs *are* inundative densities, that is, giving rise to those densities even without the occurrence of phage population growth. The question of interest at this point is not so much whether bacteria existing within clonal clumps might contribute to a lowering of MECs given active phage replication *in situ*, as that has already been argued in Sections 3.2. through 3.5., but instead what might represent the actual context of such lowering. To address that question, I briefly consider the use of phages as biological control agents of foodborne pathogens in foods (Section 4.2). I then provide scenarios for how such biocontrol could take place especially at sub-millimeter scales within foods (Section 4.3), with additional issues presented in Section 4.4. Further quantitative consideration of phage treatment of foods can be found in the Appendix. Phage treatment of microbiomes more generally is considered as well, in Section 4.5.

### 4.2. Phage-mediated biocontrol of foodborne pathogens

Contamination of food products with human pathogens, along with food-spoilage organisms, is a problem which has been dealt with through the ages by a variety of means including better sanitation, competition with non-pathogenic organisms (within fermented foods), application of chemical preservatives (e.g., salt), physical processing (i.e., heat and canning), or use, during various stages of processing, of additional antimicrobial substances (such as chlorine or organic acids). The latter both can and does include phage application at various steps going from farm to fork [18],[53],[54] (see the Appendix for a list of additional reviews considering especially post-harvest phage-mediated control of bacterial pathogens in food). Contamination with foodborne pathogens ideally will be relatively slight due to a combination of sanitation and not allowing substantial opportunity for bacterial replication during food storage. Indeed, the goal for pathogen-contaminated foods is not one of eliminating pathogens from highly contaminated products, but instead one of reducing pathogen numbers given (ideally) a potential for only relatively small pre-treatment pathogen loads. As such, the ability of foodborne bacterial pathogens to globally support *active* phage treatment—following application of phages to foods to control these organisms—should universally be viewed as slight. This low potential to support globally active treatment is a result of proliferation thresholds of target bacteria, as required simply to sustain phage populations (Section 3.2), at most being barely reached in otherwise comestible foods. Foods which are to be phage treated in other words ideally will not be contaminated with so many problematic bacteria that a globally active treatment would be possible.

It is important to keep such subtleties in mind when delving into the phage-mediated biocontrol of food literature (Appendix). In particular, there are experiments in which, rather than allowing at most only minor bacterial replication to occur, instead permit substantial bacterial replication to take place, particularly post phage application. This can be seen especially when large initial inocula of bacteria are used in combination with considerable lengths of bacterial growth-permissive incubation, such that ultimately phage replication to somewhat higher densities across an environment, potentially globally giving rise to inundative phage densities, becomes at least theoretically possible. In light of such experiments, it is important to keep in mind that the goal in phage-mediated biological control of bacteria in foods should be one of preventing such bacterial growth rather than phages impacting overgrown bacterial populations after such growth has occurred. The important issue, that is, is not so much the achievement of decreases in numbers of bacteria present relative to untreated controls but instead to both achieve and maintain reductions in numbers of bacteria to levels which in absolute terms are quite low.

From Hagens and Loessner [18], p. 63: “Any realistic experimental setup would therefore mean employing low levels of bacteria. …when investigating an antimicrobial that has the ability to kill the target organism as a post-lethality treatment, that is, following a bacterium-killing processing step such as cooking, then lower or even very low artificial contaminations are preferable.” In addition, “Abusive (high) temperatures providing optimum growth conditions for the undesired contaminants may occur either during storage at home or even at the retailers. Therefore, efficacy testing at higher than normal storage temperature has its merits, but perhaps in parallel with testing under recommended storage conditions.” Thus, it is important to both be aware of and to distinguish among different experimental approaches, those associated with treatments during industrial food production on the one hand, in which only relatively low numbers of bacterial contaminants are expected and globally active treatment is not likely, and, on the other hand, those which explore much more abusive conditions in which high numbers of bacteria along with globally active treatment at least possibly may be observed. It is predominantly the former circumstance, as potentially involving locally active treatment rather than solely passive treatment, and certainly not globally active treatment, which is under consideration here.

In considering the phage-treatment of solid or semi-solid food literature (see the Appendix), there appears to be a tendency to perform experiments in which passive treatment predominates early on, but bacterial grow-back nonetheless often occurs, with little evidence of subsequent active treatment-type phage impact. In addition, phages are generally applied soon after bacterial challenge. Consequently, formation of bacterial clumps prior to phage application likely at best is minimal in perhaps most phage-mediated biocontrol of foodborne bacteria experiments. In the real world, to the extent that substantial, especially unrefrigerated delays can occur between contamination of solid or semi-solid foods and phage application (i.e., many hours, e.g., such as following contamination during harvesting), then clumping would be expected and locally active treatment could be relevant. As noted, such scenarios in which locally active treatment would be likely to occur are not, however, typically incorporated into phage-mediated biocontrol of foods experimental studies. Instead, it is common for experiments to optimize efficacy, that is, with simpler to-treat scenarios (i.e., phage application soon after bacterial challenge without bacteria having much opportunity to replicate in association with foods prior to phage treatment) rather than emphasizing more challenging, potentially more real-world-like scenarios. Furthermore, emphasis tends to be placed on achieving statistically significant differences between phage-treated and not-treated outcomes rather than reducing bacterial numbers to acceptable levels and then keeping bacteria at those levels over reasonable time frames. That is, treatment scenarios tend to be insufficiently challenging and study end points tend to arrive before reasonable treatment efficacy has been achieved.

### 4.3. Scenarios for clumping-impact on eradication of foodborne pathogens

What is of interest here is especially the contamination of foods with unwanted bacteria which in the course of processing and storage have had some opportunity to replicate and thus, especially for more solid or semi-solid foods, have potentially formed into highly localized, clonal bacterial clumps. These clumps may consist of only a relatively few cells, e.g., two, four, or eight, i.e., rather than just one. The premise, however, is that as the total bacterial load found in these foods will be both calculated and impact consumers on a per-cell rather than per-clump basis, then that load, for example, may be eight-fold higher given three rounds of bacterial binary fission post contamination versus no such *in situ* bacterial replication. Two scenarios are considered in this section. These are treatment of bacterial clumps within foods which relatively lack spatial structure (that is, which are at least moderately well mixed and therefore within which globally active treatment is more possible, but clumping less likely) and treatment of foods which possess spatial structure, i.e., spatial structure as due to the presence of food matrix (in which globally active treatment is somewhat less possible due to limitations on phage movement, but bacteria replication into clumps more likely).

Should clumps form within moderately well-mixed environments—there for example forming as cellular arrangements—then the rate of phage access to these clumps should be a function especially of phage numbers. If phages are not able to productively infect once they reach these clumps, that is, produce new phage virions, then not only must every cell within a clump be inherently reachable by a provided phage, but for reasons of Poisson distributions, more than one phage must adsorb for every bacterium targeted in order to achieve 1 – e^−*M*^ levels bacteria killing. Here *M* is MOI_actual_, which is the ratio of the number of adsorbed phages to the number of targeted bacteria. For example, with on average 1 phage adsorption per bacterium, then only 63% of targeted bacteria will have been adsorbed by at least one phage and thus killed. As noted (Section 3.1.), degrees of adsorption where every targeted bacterium instead becomes adsorbed by multiple phages should be readily achieved over relatively short spans of time given the presence of roughly 10^8^ phages/ml, *in situ* concentration. A liter of somewhat fluid food thus could require 10^11^ phages in total (10^3^ ml/liter × 10^8^ phages/ml), or even 10^12^ if being conservative (see Appendix) toward substantially increasing the likelihood of eradication of phage-sensitive bacteria. This then represents a best-case treatment scenario given phages that, e.g., such as due to refrigeration, are unable to replicate in association with targeted bacteria, but with little environmental spatial structure interfering with phage access to bacteria. With sufficient environmental fluidity and dosing with sufficient numbers of only bactericidal virions, then phages should still be able to relatively easily reach and then kill all targeted bacteria, except possibly those which are shaded within bacterial clumps by other bacteria.

If *in situ* phage replication instead can occur, then multiple though somewhat related issues could come into play given bacterial clumping which can be relevant even given relatively high availability of bacteria to phages. These issues, in particular, could substantially diminish MECs, where smaller MECs generally would be considered to be a good thing, i.e., fewer phages required to achieve efficacy. Specifically: (I) The target size of clumps, as phage replication-supporting entities, should be greater than that of individual bacteria (Sections 3.3.3 and 3.5.), having the effect of increasing the rate with which phages can find individual targets. If the time during which phage adsorption may occur is limited, then this means that fewer phages will be required to reach the same number of targets over that span of time (Section 3.4.). Recall especially *Pkt* (Section 3.1.1.): For any cutting of the overall duration of phage exposure in half, then two-fold higher phage densities are required to achieve the same degree of phage adsorption, but for every doubling of target size (i.e., as impacting *k*), then half as many phages are required for the same rate of per-target adsorption (that is, *k* on a per-target basis in effect would be two-fold larger). (II) With clumping, for a given overall density of target bacteria, there are fewer overall targets (i.e., *N*/*n* total targets). If there are fewer targets then fewer phages must adsorb to achieve a given to-target multiplicity of phage adsorption. This also should allow for either reaching necessary levels of phage adsorption faster (i.e., *n*-fold fewer passive adsorptions must take place; Section 3.5.2.) or, given essentially infinite time, then there is a requirement for fewer phages to result in the same number of passive adsorptions per target, bacterial clumps versus individual bacteria (assuming, as always here, that the total number of target bacteria per volume remains constant, with or without clumping). (III) For every passive adsorption—given clumping along with some preference for adsorption by released phages to bacteria located within the same clump—then more bacteria will be eradicated than otherwise would be the case (i.e., locally active treatment). Given clumping, therefore, then (I) phages should adsorb these larger individual targets faster, (II) if phages are able to replicate once they have adsorbed, with resulting progeny virions not instantaneously diffusing away, then these phages will need to adsorb fewer targets initially, that is, passively, and (III) for every target initially adsorbed, again given a phage ability to replicate and be retained within clumps to at least some extent post lysis, then more bacteria will die.

The first scenario, as just considered, is one of clump presence within a fluid environment. There globally active treatment is more readily achieved for a given density of phage replication-supporting bacteria and this is due to greater phage-virion freedom of movement, that is, phages generated in one location within an environment can relatively easily reach bacteria found in somewhat different locations within the same environment. At the same time, however, clump formation is less likely. The second scenario assumes instead that bacteria are growing as clumps in association with spatial structure that can somewhat interfere with phage movement, but nevertheless does not fully interfere with that movement. That is, in this second scenario phages are assumed to be neither irreversibly trapped by food matrix nor otherwise rapidly inactivated, as those circumstances would greatly diminish the efficacy of treatments with or without bacteria replicating into clonal clumps. Thus, at least some movement of functional virions, via diffusion, can occur. The result nonetheless is that globally active treatment is less easily achieved for a given density of target bacteria—new virions generated in one location can less readily reach bacteria found in other locations—and bacteria generally are less easily reached even by dosed phages. Bacteria otherwise, however, can more readily replicate into clumps, i.e., as microcolonies.

Given these assumptions—that is, reasonable levels of virion stability in combination with substantial environmental spatial structure, yet not so much spatial structure as to completely preclude virion movement—then we can envisage a phage-treated environment in which some targets are found quickly whereas, within the same environment, phage movement to the vicinity of other targets will take more time and/or require more phages. With a clonal clumping by bacteria and given phage replication following their acquisition of clumps, however, then the number of bacteria eradicated per passive adsorption will be that number of bacteria making up individual clumps, but potentially no more than that number given spatial structure-imposed limitations on phage movement between clumps. With sufficient spatial structure, that is, then individual bacterial clumps may be viewed as equivalent to a series of physically separate bacterial sub-cultures. Given active treatment, then only one phage need reach each sub-culture to eradicate that sub-culture, whereas without active treatment, i.e., without *in situ* phage replication, then many more than one phage for each bacterium present must reach each individual, not necessarily easily reached sub-culture to achieve similar levels of bacteria killing. Thus, even should phages not be able to overwhelm target bacteria with high numbers of adsorptions, with clumping they still can substantially reduce numbers of bacteria, i.e., with *n* times as many bacteria removed per passive adsorption with clumping (given *n* cells per clump) versus without clumping. This advantage will be present, however, only if locally active treatment is possible, that is, only if phages are able to replicate *in situ* following their encounter with target bacteria. By contrast, with less spatial structure then the potential for each passive adsorption to reach more than *n* bacteria can be greater, that is, with “sub-cultures” in effect being less physically separated.

Clumping thus should increase rates of phage adsorption to targets, should reduce the total number of targets (number of bacteria which need to be passively adsorbed), and should increase the total number of bacteria killed per target passively adsorbed. With smaller clumps, then overall effects of clumping would not be as great. Still, it seems clear that it should not take much clumping to result in fairly large decreases in MECs or, and likely of greater value, greater levels of biocontrol for a given phage density applied. As has been noted here repeatedly, however, such effects are possible only if phages are able to generate new bursts over reasonably short time frames while in association, *in situ*, with target bacteria. Active treatment, in other words, always requires reasonably robust phage abilities to replicate, while purely passive treatment, also always, requires higher phage doses than does active treatment, and potentially substantially higher phage doses. With a combination of relatively low overall bacterial numbers, substantial limitations on phage movement, and bacterial clumping, then these active effects should be seen especially over only smaller spatial scales, i.e., sub-millimeter. The degree of phage performance required to effect such locally active treatment may not be as great as required to increase phage densities over larger spatial scales, i.e., as required for globally active treatment (Section 3.4.2.), but so too MECs for locally active treatment likely will be greater than for globally active treatment, while for passive treatment highest of all.

### 4.4. Forcing passive treatment and its consequences

Passive treatment on the one hand can mean simply applying sufficient numbers of phages that *in situ* phage population growth is not necessary to achieve inundative densities. On the other hand, passive treatment can be obligatory if employing phages or conditions which in fact preclude *in situ* phage population growth, that is, for which passive treatment in a sense is forced. As noted, when passive treatment is required or effected, then this will require dosing with greater numbers of phages (as MECs) than when active treatment is possible. Given spatial structure, i.e., in which phage movement is substantially reduced, then globally active treatment may not be possible, and this can be true even if in the absence of spatial structure, i.e., as in well-mixed broth, bacterial densities might have been sufficient to support a globally active treatment. Under these same circumstances, locally active treatment nevertheless may still allow a leveraging of the impact of a given phage dose, i.e., to greater than those levels of bacterial eradication which could have been achieved given obligately passive treatment while using the same phage dose. MECs given locally active treatment, in other words, should be lower than MECs for purely passive treatment.

An obligatory passive treatment can be observed under a number of scenarios. One involves substantial spatial structure in which bacteria in fact are not clumped, i.e., as appears to be the case for many experiments involving phage-mediated biocontrol of foodborne bacteria within solid or semisolid foods (Appendix). Here, even if phage replication is possible, then spatial structure, particularly given relatively low bacterial densities, likely will interfere with the potential for newly released phages to reach other, randomly dispersed bacteria. Thus, phage impact will be mostly limited to that following passive adsorptions rather than including substantial numbers of active adsorptions as well. The alternative scenario involves the use of phage or phage-like entities which are not capable of productively infecting bacteria. This can include use of so-called tailocins [55] (which are phage tail-like bacteriocins), phages which are engineered to not produce virion progeny [46], phages whose productive host range does not overlap with target bacteria even if its bactericidal host range does [28], bacteria which are treated with agents which result in a knocking out of their ability to reproduce but not their ability to bactericidally infect [56] (such as ultraviolet radiation), or use of conditions which interfere with phage replication such as refrigeration. All of these mechanisms can inhibit locally active treatment, and globally active treatment as well. Thus, we can predict that greater phage densities in these cases may be required to effect adequate levels of bacteria killing, that is, if forcing passive treatment versus allowing active treatment.

A more subtle consequence of forcing passive treatment may be found in terms of phage penetration to target bacteria. As noted, within clumps bacteria may shade each other from phage attack. This can take the form of delaying phage passive adsorption to bacteria or instead could outright block passive adsorption to certain individual bacterial cells. Without *in situ* phage replication, then there can be no active adsorption to such otherwise difficult to reach bacteria. Forced passive treatment thus could be less effective in terms of phage penetration to target bacteria than active treatment, a concept mentioned above in terms of “active penetration”. Even more subtle is the treatment of bacteria under conditions which could allow active treatment, but with conditions then switched to those which instead would inhibit active treatment, particularly refrigeration following phage application. In this case, we can speculate that locally active treatment, though in principle possible, still may be less effective in its impact. Indeed, if refrigeration is fully inhibiting of phage replication and initiated prior to the completion of at least one phage latent period, then in effect passive treatment will have been forced despite an opportunity for active treatment. This, however, would be less true if refrigeration did not completely block the release of already intracellularly formed virion progeny or instead if such latent-period completion could still occur once inhibiting conditions were reversed, such as upon food preparation for consumption. At the same time, removal of inhibiting conditions could allow bacterial growth, particularly of bacteria which have not yet been reached by phages. Active treatment, even if only local, though potentially more effective than passive treatment, nonetheless likely requires greater thought and experimental exploration to effectively implement versus purely passive treatments.

### 4.5. Microbiome modulation using phages

Technically, a microbiome as microbiota can consist of all of the microorganisms making up an environment, even given the occurrence of undesired perturbations of that environment. Thus, biocontrol of food-contaminating, undesired bacteria using phages can be described as microbiota modulation as too can the phage therapy of bacterial infections of, for example, animals such as ourselves. Typically, however, microbiomes, as collections of organisms or their genomes inhabiting a given environment, are considered less in terms of acute perturbations and more so in terms of what organisms are persisting including organisms which are not necessarily easily identified as contributing to a disease state. Nevertheless, increasingly microbiome members are being associated with various chronic perturbations of health, leading to a broader application of the idea of microbiota modulation [57]. One approach to addressing issues of presence of undesired microorganisms is selective depletion of those organisms without otherwise modifying microbiomes [58], i.e., microbiome editing, which in principle can be addressed using phages [59]. As noted, phages similarly may be employed to selectively reduce numbers of specific, undesirable bacteria in foods.

As has been discussed throughout this article, we can distinguish in terms of phage-mediated biocontrol of bacteria at least three situations. First are those circumstances which are able to support phage replication to inundative densities across environments, thereby allowing a globally active treatment. Second are those circumstances in which only passive treatment is possible. Third are those in which locally active treatment is possible but not globally active treatment. For a given type of bacterium and conditions, generally we can distinguish these environments in terms of a combination of densities of those bacteria which can support the replication of specific phages along with the rates of movement that phages are capable of achieving between bacteria. Above a particular density threshold, bacteria will be sufficiently abundant such that movement between bacteria is rapid enough to support a globally active treatment, i.e., as equivalent to a critical density of bacteria that is able to sustain a phage-reproduction chain reaction. Below that threshold then passive treatment will be necessary because *in situ*-generated phages either will tend to decay somewhat faster than they can reach new bacteria or such movement will be slower than is desirable from a biocontrol perspective. In between, of course, are when bacteria are found in clonal clumps which individually possess bacterial densities that are sufficient to support active treatment, but bacteria found external to these clumps are insufficiently prevalent to be reached with high likelihood by *in situ*-generated phages either in a timely manner or instead faster than those phages decay. For a typical acute infection, however, bacteria can be present at sufficient densities across treated environments that globally active treatment may be possible. This is because such infections can involve substantial overgrowths at the site of infection of a single or at most a few bacterial strains [60]. So too if one is targeting major bacterial components of a sufficiently dense as well as actively metabolizing microbiome, then globally active treatment should be possible, that is, as across the volume or area that is being treated.

The issue that is of chief consideration here is what happens when instead an only minor component of a microbiome is targeted such that densities of target bacteria are not inherently sufficiently high or phage movement sufficiently free to support globally active treatment? One answer to that question is obvious, and that is to supply sufficient numbers of phages to conservatively achieve passive treatment. There are at least three issues that will come up regarding the latter assertion, however. The first is the question of just how many phages may actually need to be applied to in fact achieve adequate and especially lasting microbiome modification, e.g., a sustained 10^8^/ml? 10^9^/ml? More? The second is the question of whether truly passive treatment can even be achieved, that is, treatment success even if phages are unable to replicate *in situ*, e.g., as particularly can concern the issue of whether successful phage penetration to passively adsorb all target bacteria is even possible. The third point is related to points one and two, and that is whether phage replication *in situ* in fact can contribute to greater treatment success even if there are insufficient numbers of bacteria to support a globally active treatment.

As with treatment of foods, these questions lead to two more considerations, and that is whether or not target bacteria are present as clumps and also whether or not target bacteria in fact can support phage replication *in situ*. Thus, given presence of target bacteria within clonal clumps in combination with an ability of phages to replicate to a sufficient extent once those clumps have been reached, then a locally active treatment may be possible. For the sake of selectively removing bacterial members of microbiomes, even if globally those bacteria are not present at sufficiently high densities, it therefore can be relevant to experimentally make sure that sufficient phage numbers are applied, that those phages are able to reach some approximation of all targeted bacterial clumps, and also that these phages have a potential to display not just bactericidal infections once bacteria have been reached but phage-productive ones as well. In this way, the potential for phages to remove all of the bacteria associated with bacterial cellular arrangements or microcolonies literally may be amplified, even if the ability of resulting amplified phages to reach other bacterial clumps is low. The degree of *in situ* phage amplification required to eliminate individual bacterial clumps, however, may not be as great on a per-infection basis as that required to achieve globally active treatment.

At the same time, phages must be dosed at levels which are sufficient for them to physically reach an approximation of all bacterial clumps, but if globally active treatment is possible then dosing leeway should be much greater. That is, MECs given globally active treatment should be somewhat lower than MECs given locally active treatment, which in turn should be lower than MECs given only purely passive treatment. Or, in other words, though we likely will not be able to achieve microbiome editing in many cases using delivered phage densities which are substantially lower than inundative densities (e.g., 10^5^ virions/ml, *in situ*), given bacteria formation of clonal clumping it at the same time may not be necessary to supply conservatively calculated inundative densities as MECs (e.g., 10^9^ virions/ml, *in situ*). Or, rather, less conservatively calculated MECs for passive treatment may achieve greater bacterial eradication given localized phage propagation *in situ* than may be achieved without such propagation.

In short, while researchers should be aware that active treatment generally will require densities of target bacteria which are relatively high (e.g., 10^6^ bacteria/ml, or even greater densities given relatively small phage burst sizes), at the same time they should not disregard the utility of a phage potential to replicate *in situ*, that is, just because there are insufficient bacteria present to support across-environment phage population growth to inundative densities. At the same time, a phage potential to locally amplify its numbers, also *in situ*, should not be used as an excuse to substantially reduce the numbers of phages that are applied, i.e., since locally active treatment requires, at a minimum, that a substantial fraction of bacterial clumps in fact be passively adsorbed. To explore these issues experimentally, that is, to determine the utility of using higher phage doses versus leveraging phage replication as antibacterial strategies, it will be necessary not only to employ realistic model systems, i.e., which can support bacterial replication into sparsely dispersed clumps given an otherwise *in situ* propensity for bacteria to do so, but also a means of inactivating phages in terms of their ability to produce new virions while not impacting their potential to infect bactericidally, e.g., such as via UV irradiation of virions [56].

Distinguishing active from passive treatments can involve observation of greater bacterial survival, i.e., less killing, if using equivalent numbers of bactericidal but not productively infecting phages versus productively infecting phages (keeping in mind that titers of phages for such experiments should be determined using killing titer assays [61],[62],[63] rather than as based solely on the evaluation of phage numbers prior to inactivating treatment). Insubstantial bacteria killing using *in situ* replication-competent phages, unless higher phage densities are delivered (e.g., 10^8^ or even 10^9^ virions/ml final concentration rather than 10^5^), would suggest an inability of environments to support a globally active treatment. Substantial increases in bacterial eradication which is phage replication dependent under conditions where globally active treatment otherwise is not observed to occur would be seen as an indication of a locally active treatment. The possibility of such locally active treatment should be considered whenever removal of especially relatively minor microbiome components, via phage treatment, is being explored.

## 5. Conclusions

If densities of target bacteria are sufficiently high, roughly at least 10^6^ bacteria/ml (and certainly not substantially lower), then the potential for those cultures to support a globally active phage treatment can be reasonably robust. This is due to an ability of phages to replicate to inundative densities across environments (roughly at least 10^8^ phages/ml) given the presence of sufficient densities of metabolically active host bacteria. At lower bacterial densities, or given sufficient barriers to virion movement, then even if net phage population growth is still possible, and even if bacterial densities exceed thresholds for ongoing phage proliferation, the potential for phages to reach inundative densities through phage population growth can be diminished. It is possible to address this issue by reaching inundative densities through dosing alone, that is, via a purely passive treatment. Given reasons to apply fewer phages than fairly overwhelming doses, however, then it likely is helpful that the association of bacteria into physically linked clonal groups, here called clumps, could leverage phage replication towards a locally active treatment even if globally active treatment is not achievable.

Targets of locally active treatment would be especially bacteria that have had an opportunity to replicate to some degree, though not overwhelmingly so, prior to the commencement of phage treatment. This is particularly such that this bacterial replication gives rise to small clusters of bacteria, that is, microcolonies or cellular arrangements, without necessarily replicating to such high densities as to grossly contaminate foods, for example. Indeed without necessarily replicating to sufficient densities to support globally active treatment. The conclusion, minimally—and especially when working with bacteria which are inherently sensitive to applied phages—is that pharmacological models which are based on an assumption of a lack of clonal associations among bacteria may provide an underestimation of the potential for phages to serve as biological control agents of bacteria, perhaps particularly given post-harvest biological control of foodborne pathogens. Such utility, however, requires a phage potential for some population growth during treatment, though not necessarily as much productivity as can be required to effect an active treatment more globally.

Using phages which are able to replicate on a wider diversity of potential target bacterial strains, rather than displaying just bactericidal activity, thus could be helpful towards biological control of foodborne bacteria, or more generally towards the modification of microbiomes via phage-mediated editing out of less prevalent but nonetheless problematic bacteria. This furthermore could be true even if the degree of phage replication, as a fraction of the total number of phages applied, is relatively tiny and thereby difficult to actually measure in the course of treatments. In practical terms, this usefulness points to utilities of explicitly selecting for phages, in the course of phage isolation, as well as subsequently characterizing phages in the course of host-range determination, for an ability to produce virions under expected treatment conditions. That is, a demonstration, for a diversity of potential target bacteria, either of plaque forming ability or, within broth, a capability of displaying population growth while experimentally striving to mimic the chemical and physical aspects of to-be-treated environments. It also suggests that intentionally disabling the ability of phages to replicate, even as bactericidal activity is retained [56], could have efficacy ramifications given bacterial growth into clumps prior to the initiation of phage treatment.

Hagens and Loessner [18] list 10 phage properties which are desirable for their use as antibacterial agents in foods. These are: (I) broad host range, (II) not temperate (no lysogeny), (III) propagation during production on hosts which themselves are not pathogenic, (IV) completely sequenced, (V) non-transducing (particularly generalized transduction), (VI) lack of encoding of human-harmful genes, (VII) otherwise lacking in toxic effects, (VIII) generally recognized as safe (GRAS) regulatory approval, (IX) stability, and (X) potential to be manufactured in large quantities. To this list, especially given target bacteria presentation as clonal clumps, the utility of a phage ability to productively infect bacteria *in situ* during biological control should be considered as well. That is, a phage ability to replicate *in situ* should be viewed as a potential improvement over antibacterial strategies which instead are purely passive, even despite expectations that bacterial numbers within foods overall will be too low to globally support active treatment. Locally active treatment will not be achievable without active phage replication, nor even possible absent clonal bacterial clumping, but given an ability of target bacteria to replicate into clonal clumps prior to treatment, and despite otherwise low densities of those bacteria globally, employing phages which themselves are able to replicate over the course of treatments, as well as supplying conditions which can support that replication, could provide extra margins of antibacterial efficacy.
