# Supplementary material for: Active bacteriophage biocontrol and therapy on sub-millimeter scales towards removal of unwanted bacteria from foods and microbiomes
Source: AIMS Microbiol. 2017 Aug 4;3(3):649–88. doi: 10.3934/microbiol.2017.3.649 (PMC6604992; doi:10.3934/microbiol.2017.3.649)
Supplement: Supplementary file 1 [file microbiol-03-03-649-s001.pdf]

AIMS Microbiology

http://www.aimspress.com/journal/microbiology

AIMS Microbiology, 3(3): 649-688.
DOI: 10.3934/microbiol.2017.3.649
Received: 16 May 2017
Accepted: 19 July 2017
Published: 04 August 2017

Research article

Active bacteriophage biocontrol and therapy on sub-millimeter scales towards removal of unwanted bacteria from foods and microbiomes

Stephen T. Abedon *

Department of Microbiology, the Ohio State University, 1680 University Dr., Mansfield, OH 44906, USA

* **Correspondence:** Email: abedon.1@osu.edu; Tel: +1-419-755-4343.

1. Appendix: Quantitative Overview of Phage-Mediated Biocontrol of Bacteria in Foods

Recently published is a guide to reporting on phage therapy or phage-mediated biocontrol studies [1]. From the perspective presented there, along with as presented in [2], the overall goal of this Appendix is to explore the potential for locally active treatment (see below and main text) in light of published studies on phage-mediated biocontrol of especially solid or semisolid foods. Because a comprehensive review of this literature is beyond the scope of this article, I limit myself to a single year, 2016. A much more extensive list of phage therapy and phage-mediated biocontrol publications can be found at [3,4], and I invite readers to consider these other phage therapy and phage-mediated biocontrol studies with similar scrutiny as found here. A report critically reviewing phage use as antibacterial agents specifically in food also is available [5], and numerous reviews on or overviews of the subject of phage-mediated biocontrol of foods exist [6–43].

As this is a quantitative overview, a reoccurring theme is the calculation of multiplicity of infection (MOI), and this is done in terms of both MOI_{input} and MOI_{actual}. MOI_{input} is the ratio of added phages to bacteria while MOI_{actual} is the ratio of adsorbed phages to bacteria [44].

MOI_{input} is the easier to calculate as it consists simply of the number of phages that have been applied to bacteria-containing environments divided by the number of bacteria which are present in those environments at the point of phage application. This ease of calculation, however, comes with the caveat that both the number of phages added and the number of bacteria present at the point of phage application are not always known nor obvious. MOI_{input} also is neither a terribly useful nor meaningful measure [44], but nonetheless when dosing has been reported simply as MOIs, that is,

without qualification, then these typically should be assumed to represent MOI_{input}s [45], unless of course indicated otherwise.

If phage doses in titers are not presented within an article, then dosing numbers often can be calculated by multiplying target bacterial density by MOI as MOI_{input}. Phage titers used for dosing, especially as PFUs/ml, however, are not necessarily easily discerned from non-titer-based dosing data. It is important to stress, though, that few parameters are more relevant to phage-mediated biocontrol than the titer of phages present within applied formulated products. Therefore, this value ought to be unambiguously provided in phage therapy/biocontrol reports. Nonetheless, if not provided then phage titers often (though not always) can be calculated based on MOI_{input} information.

MOI_{actual} is measured based on the number of bacteria present within treated environments in combination with the number of phages which have been found to have adsorbed. This value can be estimated from log reductions in bacterial concentrations as $-\ln(10^{-\log\ reduction})$ [46,47,48]. Thus, log reductions of 1, 2, 3, 4, and 5 (10-fold, 100-fold, 1000-fold, etc.) can be estimated as corresponding to MOI_{actual}s of 2.3, 4.6, 6.9, 9.2, and 11.5 adsorbed phages per targeted bacterium, respectively. Here, in calculating log reductions, I focus on reductions from those bacterial densities that were present at time zero, that is, immediately prior to initial phage application, and this is rather than relative to bacterial densities that are found at a given time point as associated with phage-less controls. It is important to point out that log reductions from original bacterial densities appear to be rarely reported in this literature so therefore this value, where necessary, has been approximated, and these approximations have been made simply by eye. It should be obvious in reading this Appendix, however, that imprecisions resulting from this estimation approach will tend to have little impact on overall conclusions. Nonetheless, it would be useful were authors to routinely report such values, i.e., fractional, phage-associated reductions in bacterial densities from original, time-zero bacterial densities (that is, as present just prior to phage application).

Based on this various quantitative information as well as additional details of experimental design and observations, here I seek insight into the potential within experiments for passive treatment, globally active treatment, or locally active treatment. These, as covered in greater detail in the main text, are phage treatment that does not require phage replication *in situ* but instead only phage bactericidal activity (passive treatment), phage treatment in which *in situ* phage replication is required as generally resulting in substantial increases in phage titers across treated environments (globally active treatment), and phage treatment in which *in situ* phage replication is required, but where the impact of that replication on phage densities occurs only over small spatial scales, i.e., less than one millimeter distances and approximately the size of a bacterial cellular arrangement or microcolony (locally active treatment). The latter, i.e., bacterial cellular arrangements or microcolonies, I collectively describe as bacterial "clumps".

Discussions of individual articles I present in first-author alphabetical order. Where appropriate, the degree of insight which either can or cannot be ascertained from the information provided by individual studies is indicated. The Appendix ends with a number of suggestions on how it might be possible to improve upon approaches towards characterizing the use of phages as antibacterial agents within foods (Section 1.18). Important abbreviations include CFU (colony-forming unit) and PFU (plaque-forming unit).

1.1. Baños et al.

Baños et al. [49] treated raw fish (hake and salmon) or smoked fish (salmon) with the anti-*Listeria monocytogenes* phage, P100, with a subsequent 4 °C incubation. A challenge of 10^3 CFU/cm^2 was spray applied which was followed by air drying (30 min; temperature not specified) and then a spray-applied phage dose of 2.3×10^7 PFU/cm^2 (phage density within the phage formulated product not specified). Approximately 1 log reductions in CFUs from original challenge densities were observed after 1 day of incubation for raw fish and less than a 1 log reduction after 1 day for smoked salmon, but a 1.5 log reduction for the latter at day 5. MOI_{input} was 2.3×10^4 while the observed CFU reductions are consistent with MOI_{actual}s of approximately 2.3, less than 2.3, and 3.5, respectively, implying that most applied phages did not reach bacteria. Impact of greater phage doses was not determined though if all bacteria were equally reachable by phages then we can speculate that a phage dose of approximately 10^8 PFU/cm^2, i.e., a four-fold increase, could have been sufficient to achieve an approximation of complete bacterial eradication (i.e., as achieving a predicted MOI_{actual} of 10). If that were insufficient to eradicate all targeted bacteria then experiments employing ten-fold greater numbers, i.e., resulting in 10^9 PFU/cm^2, could have been attempted as a bacteria-killing positive control. The need for greater killing is particularly the case given the approximately 1- to 2-log increases in bacterial densities post phage-associated reductions in bacterial densities. Though untreated bacteria also increased their numbers, by approximately 2 to 3 logs over seven or 30 days at 4 °C, post challenge for raw fish and smoked fish, respectively, there is no evidence of *in situ* bacterial replication prior to phage application, and pre-treatment bacterial replication is especially unlikely since a stationary phase bacterial inoculum was used. There does not otherwise appear to be evidence of either globally or locally active treatment, thus suggesting that only passive treatment occurred, especially early on, i.e., during the first day of incubation.

1.2. Boyacioglu et al.

Boyacioglu et al. [50] treated spinach with the anti-*Listeria monocytogenes* phage formulated product, ListShield^{TM}, with a subsequent 4 °C or 10 °C incubation. A challenge of about $10^{4.5}$ CFU/cm^2 was spray applied which was followed by air drying (20 min; temperature not specified) and then a spray-applied phage dose of 10^6 PFU/cm^2 (10^7 PFU/ml as applied). Approximately 3 log reductions in CFUs from the original challenge densities were observed after 1 day of incubation. MOI_{input} was approximately $10^{1.5}$ (about 30 phages/bacterium) while the observed CFU reductions are consistent with an MOI_{actual} of approximately 7, implying that most applied phages did not reach bacteria. Impact of greater phage doses was not determined though if all bacteria were equally reachable by phages then we can speculate that a phage dose of less than 2×10^6 PFU/cm^2 could have been sufficient to achieve an approximation of complete bacterial eradication (i.e., as achieving a predicted MOI_{actual} of 10 or more). If that were insufficient to eradicate all targeted bacteria then experiments employing ten-fold greater numbers, i.e., ~2×10^7 PFU/cm^2, or even simply 10^7 PFU/cm^2, could have been attempted as a bacteria-killing positive control. The need for greater killing is particularly the case given approximately 2-log increases in bacterial densities post phage-associated reductions in bacterial densities at 10 °C. Though untreated bacteria increased their numbers by approximately 1 log over 14 days at 10 °C, post challenge (versus about 1 log growth at 4 °C), there is

no evidence of *in situ* replication of bacteria prior to phage application, and pre-treatment bacterial replication is especially unlikely since a stationary phase bacterial inoculum was used. There does not otherwise appear to be evidence of either globally or locally active treatment, thus suggesting that only passive treatment occurred, especially early on, i.e., during the first day of incubation.

1.3. El Haddad et al.

El Haddad et al. [51] treated cheddar cheese during production with an anti-*Staphylococcus aureus* phage cocktails, with a subsequent 4 ℃ incubation. Prior to curd formation a challenge of 10^6 CFU/ml was added which was followed by phage doses of 1.5 × 10^7, 4.5 × 10^7, or 15 × 10^7 PFU/ml. Substantial declines in bacterial densities do not appear to have occurred prior to curd formation, suggesting a lack of passive treatment. Declines in bacterial densities, however, were observed following coagulation and cheddaring steps, with more rapid declines seen given application of higher phage densities. As bacteria were little affected by phages prior to coagulation, exhibited replication or at least increases in numbers over the course of incubation absent phages (particularly during the coagulation step), and may, as staphylococci, have displayed some clumping prior to challenge, i.e., as cellular arrangements, we can speculate that some degree of locally active treatment may have occurred prior to the 4 ℃ incubation. This suggestion is obscured, however, by the ability of all three phage treatments to result inexplicably in reductions in bacterial densities to undetectable levels during ripening at 4 ℃ (when phage replication presumably could not occur) as well as a lack of demonstration of phage replication *in situ*, though the observation of an increase in numbers of CFUs when phages were not applied, if indeed that is due to bacterial replication (versus a gradual separation of otherwise clumped bacteria), could be consistent with a potential for phage replication *in situ*, perhaps particularly as seen during the coagulation and cheddaring steps, that is, prior to cooling to 4 ℃. Thus, in principle locally active treatment could have occurred, but at best this is speculative for this system as presented. As phage concentrations were not determined during cheese production with *S. aureus*, it is difficult as well to rule out globally active treatment.

1.4. Galarce et al.

Galarce et al. [52] treated milled, processed meat products (barbecue sausage, chicken sausage, Italian sausage, and turkey ham) with an anti-*Salmonella enterica* Enteritidis phage cocktail, with a subsequent 4 ℃ or 18 ℃ incubation. A challenge of 10^3 to 10^5 CFU/g—as appears to be experimentally determined, though according to the reported methods instead approximately 10^2 or 10^3 CFU/g—was applied to homogenized meat product which was followed after two hours of room temperature incubation by 10^8 to 10^{10} PFU/g (these numbers are as based on the reported MOI_{input}s of a consistent 10^5 and experimentally determined bacterial densities on day 0, though in terms of reported methods 2.5 ml of either 10^8 or 10^9 PFU/ml were diluted to 30 g implying phage concentrations instead of roughly 10^7 to 10^8 PFU/g, depending on meat product treated). Approximately 0 to 1.5 log reductions in CFUs from original challenge densities were observed after 3 days of incubation. Though reported MOI_{input}s are 10^5, the reductions observed are consistent instead with MOI_{actual}s of at most 5, implying that most applied phages did not reach bacteria. Based

on Methods-calculated phage densities, it is seems likely that consistent *in situ* phage densities—that is, keeping phage titers constant going from experiment to experiment rather than adjusting MOI_{input}, i.e., see [53]—of at least two-fold greater PFUs than what was actually applied would have been a preferable dosing approach and ideally experiments employing at last 10^9 PFU/g would have been attempted as a bacteria-killing positive control. Especially at 18 ℃, bacteria were able to replicate, increasing in one experiment by 5 log. To the extent that treated foods were sufficiently solid, then it is at least possible that small microcolonies were able to form prior to phage application, along therefore with locally active treatment, though it is difficult to ascertain how this may have affected outcomes. In some experiments there is indication of increases in phage densities over time, though this oddly occurs at 4 ℃ but not at 18 ℃, so it is difficult to conclude that globally active treatment occurred. Overall, it seems like that predominantly passive treatment was observed.

1.5. Gencay et al.

Gencay et al. [54] treated Turkish raw meatballs with an anti-*Escherichia coli* O157:H7 phage, M8AEC16, with a subsequent 4 ℃ or 22 ℃ incubation. A challenge of approximately 10^2, 10^4, or 10^6 CFU/g (actual reported values were $10^{2.81}$, $10^{4.51}$, and $10^{6.30}$) was mixed into meatballs along with phage doses of about 10^9 PFU/g. At the initial time point—3 hours for 4 ℃ and 1 hour for 22 ℃—there was little difference in phage impact depending on temperature, suggesting that passive treatment was observed (that is, phage replication is not expected at 4 ℃). There were, however, differences in the fractional reduction in CFUs depending on the number of starting bacteria, with reductions from $10^{6.30}$ of log 0.7, reductions from $10^{4.51}$ of about log 1, and reductions from $10^{2.81}$ of about log 1.8. Such results could be due to the presence of bacteria, within the treated meatballs, which phages were unable bactericidally infect, with fractionally more such bacteria present given larger bacterial challenges. Such bacterial resistance could perhaps have been a consequence of delays in bacteria exiting an initial stationary phase, e.g., such as could possibly have been the case more towards the centers of meatballs (bacteria appear to have been in stationary phase when initially inoculated and therefore when they probably first contacted phages). These bacteria, I hypothesize, could have allowed phage adsorption but not subsequent phage replication, i.e., and thereby resulting in phage inactivation. Eventually, at 22 ℃, these surviving bacteria we can speculate were able to replicate within now somewhat phage-free pockets within meatballs, thereby increasing total CFUs, i.e., as measured at 24 hours. It is conceivable, given this scenario, that use of phages which were more effective in at least bactericidally infecting bacteria possessing stationary phase-like physiologies would have been more effective in reducing bacterial numbers (or, instead, given a bacterial challenge using log-phase bacteria). With the 100-plus-fold excess of phages in the high bacterial density challenge, it seems unlikely that insufficient numbers of phages was an issue at higher versus lower bacterial densities, though certainly this possibility could be easily tested. It is possible, however, that bacteria which were adsorbed by more phages given lower bacterial challenge densities displayed lower post-adsorption bacterial survival. A lack of delay between bacterial challenge and phage application suggests against the occurrence of locally active treatment, though given the extent of disruption of the food matrix in the course of phage application (i.e., kneading by hand), it is questionable whether bacterial clumps could have persisted even were there

such delay. No evidence was presented, e.g., phage titers during treatment, suggesting globally active treatment.

1.6. Hong et al.

Hong et al. [55] treated ground pork with an anti-*Salmonella enterica* (Enteritidis and Typhimurium) phage, vB_SalS_SJ_2, with a subsequent 4 °C or 21 °C incubation. A challenge of 10^7 CFU per 15 g was mixed in and this was followed with 10^8 PFU per 15 g. Starting with 6.7×10^5 CFU/g, after 24 hours at 21 °C with phage treatment there were approximately 6.7×10^6 CFU/g, that is, ten-fold more bacteria than at the start. The calculation required to determine the latter concentration is as follows (and which, as indicated further down, may not be correct): 15 g of meat were diluted into 100 ml of buffer prior to obtaining (I assume) per ml bacterial densities (their Figures 1 and 2). With *S.* Typhimurium, approximately 10^6 CFU/ml were thus detected. To obtain the number per original grams of ground pork, we multiply by 100 ml/15 g (assuming that the pork did not substantially add to the liquid volume subsequently sampled). Numbers of *S. Enterica* 24 hours post the start of phage treatment were at least 0.5 log higher. This would seem to represent a failure of passive treatment (alternatively a grow-back of phage-resistant bacteria is possible, though this was tested and did not appear to be the case). A failure of passive treatment would not be surprising given dosing with only 6.7×10^6 PFU/g of an at least semi-solid food. Such a failure of passive treatment is supported by results seen at 4 °C where, for *S. Typhimurium*, an indicated 10^4 CFU/ml were present after 24 hours without phage treatment but in excess of $10^{3.5}$ CFU/ml with phage treatment. These, according to the above calculations, represent 6.7×10^4 CFU/g and about 2.1×10^4 CFU/g, respectively, i.e., at best on average a three-fold or 0.5-log reduction, which in this case must certainly be passive treatment assuming that neither *Salmonella* nor its phage is replicating at 4 °C. Unfortunately, the calculation of 6.7×10^4 CFU/g is ten-fold off from the expected initial 6.7×10^5 CFU/g (above), which suggests that my assumption of the meaning of the figure-presented measurements presented in the paper is flawed. Thus, it is possible that rather than observing a ten-fold increase in bacterial densities with phage treatment at 21 °C, instead bacterial densities simply stayed the same. The relative concentrations determined at 4 °C nonetheless should be valid, indicating small but nonetheless statistically significant declines in CFUs. MOI_{input} was an indicated 10, but the observed relative reduction at 4 °C instead is consistent with an MOI_{actual} of at best 1, implying that most applied phages did not reach bacteria. Employing a dose of at least ten-fold more phages therefore would seem to be indicated, i.e., 10^9 PFU per 15 g, and ten-fold more than that (10^{10} PFU per 15 g) to serve as a bacteria-killing positive control. For equivalent reasons as those reported for Gencay et al. [54] (above), there is no suggestion of either locally or globally active treatment in this study.

1.7. Hudson et al.

Hudson et al. [56] treated the surfaces of raw and cooked meat with the anti-*Escherichia coli* O157:H7, UV-treated phage, FAHEc1, with subsequent 5 °C, 24 °C (for cooked meat), or 37 °C (for raw meat) incubations. Phages that are UV treated, and therefore unable to replicate, by definition are only able to effect a passive treatment. Unfortunately, resulting phage "titers" were not determined in this

study, i.e., as is possible via the employment of killing titer determinations [47,57]. As a consequence, bactericidally active UV-treated virions are at least potentially lower in number than pre-UV treated virions. A challenge of 2×10^5 CFU was dropped within 20 µl onto 2 × 2 cm pieces of cooked meat with 2×10^4 or 2×10^7 PFU then applied also within 20 µl (PFU referring to pre-UV phage activity and as corresponding to a calculated 10^6 or 10^9 PFU/ml within formulated products). With raw meat, the equivalent numbers, though with potentially different units, were 10^6 CFU/cm^2 and 3.1×10^8 PFU/cm^2, the latter at least potentially corresponding to 10^9 PFU/ml within formulated product. The 2×10^4 PFU treatment at 5 ℃, corresponding to 10^6 PFU/ml, showed no reductions in bacterial numbers over 48 hours. With cooked meat at 5 ℃—approximately 2-hours post application of phage and bacteria, using 2×10^7 PFU—there was an approximately 0.5 log reduction in bacteria with UV-treated phages and 1 log reduction with untreated phages. As noted, however, killing-titer determinations were not performed so we do not know what the actual, non-*in situ* activity of the UV-treated versus not UV-treated phages. We similarly cannot calculate MOI_{input} for UV-treated phage, though with not UV-treated phages the MOI_{input} was equal to 100 (and passive treatment even without UV treatment can probably be assumed given the 5 ℃ temperature). The 0.5 and 1 log reductions correspond to MOI_{actual}s of approximately 1 and 2.3, respectively. Unfortunately, the earlier time point (here seemingly 3 hours) was not taken for the UV-treated phages in the 24 ℃ experiments. At approximately 7 hours, where no bacterial growth absent phages was yet observed, there is approximately a 1-log decrease with not UV-treated phages and an only slightly smaller decrease with UV treated phages. This result suggests both that phage replication was not extensively occurring absent the UV treatment at this temperature or at least did not play a substantial role in impacting bacteria (since a greater advantage without UV treatment might therefore have been expected to be present) and that the bactericidal activity of the UV treated phages in fact did not decline substantially in the course of UV treatment. It is interesting, though, that this UV-treated phage activity appears to be greater relative to not UV treated at the higher temperature, perhaps implying some sort of UV-caused damage to virions which is more pronounced at lower temperatures. Not unexpected, use of seemingly higher phage densities on raw meat, though also at 37 ℃, resulted in higher levels of bacteria killing, i.e., roughly 2 logs after 25 min. Here a two-fold greater MOI_{actual} may be calculated (i.e., roughly 5) with a calculated MOI_{input} of about 300. In all of these cases it is clear that dosing with larger numbers of phages both to achieve greater bacteria-killing efficacy and to serve as a bacteria-killing positive control would be useful. In the case of raw meat, presumably at least a doubling of phage concentrations could be attempted and four or more fold greater with cooked meat, with then ten-fold greater numbers towards employing a positive control. For equivalent reasons as those reported for Gencay et al. [54] (above), there is no suggestion of either locally or globally active treatment in this study, even for the not UV-inactivated phage treatments.

1.8. Iacumin et al.

Iacumin et al. [58] treated dry-cured ham with the anti-*Listeria monocytogenes* phage, P100, with a subsequent 4 ℃, 10 ℃, or 20 ℃ incubation. Challenges of 10^2, 10^3, or 10^4 CFU/cm^2 were spread onto the surfaces of ham slices and after 30 min a phage dose was also spread on, with phage doses of 10^4, 10^5, 10^6, 10^7, and 10^8 PFU/cm^2 (phage densities within the phage formulated products

not specified). Reductions in bacteria after application of 10^8 PFU/cm^2 and 24 hours of incubation consisted of 3.7, 1.8, and 1 logs, from 4 logs (10^4 CFU/cm^2), for temperatures of 4 °C, 10 °C, and 20 °C, respectively (that is, to 0.3, 2.2, and 3.0 log remaining bacteria, again respectively). The greater killing at lower temperatures is perhaps suggestive of less re-growth of bacteria which initially were not reached by phages. Perhaps consistently, starting instead with 10^2 CFU/cm^2 there was complete eradication at all temperatures. It would be interesting to see, therefore, what the effect of applying higher phage concentrations on reductions starting with 10^4 CFU/cm^2. Especially, a consistent question with all of these studies is that of whether survival of bacteria is a consequence of application of insufficient numbers of bacteria to reach target bacteria or whether instead target bacteria that survive are unreachable regardless of how many phages are applied. Thus, application of 10^9 PFU/cm^2 to 10^4 CFU/cm^2 in this model system could help to answer that question, or even application of 10^{10} PFU/cm^2. Application of only 10^7 PFU/cm^2 appears to have substantially less impact than application of 10^8 PFU/cm^2 at 4 °C. Typically *L. monocytogenes* can replicate at 4 °C—though of interest, no increase in numbers of bacteria appeared to occur at this temperature under the incubation conditions employed—so phages too in this study may or instead may not have been able to replicate at this temperature. Phages were applied soon after bacteria in these experiments, however, suggesting a lack of bacterial clumping at the point of phage exposure and therefore little locally active treatment. MOI_{input} was 10^4 while the observed reductions of 3.7 log were consistent with an MOI_{actual} of approximately 8.5. As with previously considered studies, there is no evidence that is presented for the occurrence of globally active treatment. It is likely, therefore, that predominantly passive treatment occurred.

1.9. Kurtböke et al.

Kurtböke et al. [59] treated strawberries with an anti-family *Enterobacteriaceae* phage cocktail, with a subsequent 4 °C, 20 °C, room temperature, or 45 °C incubation. Experiments were performed targeting either naturally present bacteria or instead added challenge bacteria. The latter consisted of 10^7 CFU/ml added to surface-sterilized fruit followed by 20 min of incubation, or instead with bacteria added after phages. In either case, strawberries were then soaked in a suspension containing 10^{12} PFU/ml for one hour. Note that this density represents a sum of densities of multiple phage types with varying host ranges. This nonetheless is an impressively high density of phages to employ, and it is commendable that dosing is indicated in per ml units (as with washes generally, measure of dosing as titers presumably would be the case given real-world application). Bacterial reductions in comparison to untreated controls tended to be more than 4 logs after 48 hours of incubation. Unfortunately, it is difficult to say what bacterial loads were prior to this incubation. Numbers of bacteria remaining following phage treatment were mostly in the range of hundreds per gram of strawberry. No effort was made to determine whether or not remaining bacteria were phage sensitive, which makes it difficult to estimate MOI_{actual}s given this degree of bacterial killing as well as survival, though nevertheless log reductions predominantly would correspond minimally to MOI_{actual}s of about 10. Though in treating naturally occurring bacteria the potential for locally active treatment is possible, that is, given that bacteria could have readily replicated into clumps prior to phage exposure, the extremely high densities of phages applied in these experiments is suggestive

that such active treatment, including globally active treatment, would have been less relevant since sufficient numbers of phages should already have been present.

1.10. Ladero et al.

Ladero et al. [60] treated a model cheese during production with an anti-*Enterococcus faecalis* phage, Q69, with a subsequent 12 ℃ or 16 ℃ incubation (there is a discrepancy in the text as to which temperature was used, and this is the temperature of ripening but not necessarily that of manufacturing). A challenge of 10^4 CFU/ml along with 10^3 PFU/ml was added to milk, apparently at approximately the same time. By definition this treatment, to impact any more than approximately 10% of bacteria, must have involved active phage replication and therefore active treatment. Once cheese manufacturing was completed, and therefore was no longer in a liquid state, bacterial densities were in excess of 10^6 CFU/g. With phage presence, however, bacterial densities, per gram, were roughly unchanged from the original per ml density, but 2 log lower than without phage presence. As indicated, this would suggest a somewhat successful active treatment, and particularly so since phage densities at this point had reached approximately 10^{11} PFU/g. This active treatment, however, we can speculate occurred while the cheese was still in a liquid phase. There is, nonetheless, perhaps further declines in densities of target bacteria in the presence of phages during 60 days of cheese ripening, or at least a lack of further increases in bacterial densities given phage presence, along with further increases (about 1 log) in bacterial numbers in the absence of phages during ripening. It is possible that this decline or lack of increase in bacterial density given phage presence could be a consequence of a locally active phage impact, with *E. faecalis* cells which had escaped phage attack initially then growing into microcolonies, some of which by chance came to encounter phages, with phages then reducing overall bacterial counts within those microcolonies. These reductions could be due to partial bacteria killing per microcolony present (i.e., many phage-microcolony encounters but incomplete elimination of bacteria within each encountered microcolony) or instead could be due to encounter by fewer microcolonies but with more complete elimination of bacteria per microcolony encountered. As conducted, this experiment thus required phage replication to achieve substantial bacterial killing during cheese manufacturing, but we can speculate that further reductions could also have required phage replication especially within a more solid phase, i.e., towards effecting a locally active treatment during cheese ripening.

1.11. Lee et al.

Lee et al. [61] treated cabbage with the anti-*Escherichia coli* O157:H7 and anti-*Shigella flexneri* phage, HY01, with a subsequent 37 ℃ incubation. A challenge of 10^3 CFU was spread on 5-gram square pieces of cabbage and was then sprayed with 10^8 or 10^9 PFU (the latter as based on indicated MOIs, i.e., MOI_{input}, of 10^5 or 10^6; phage densities within the phage formulated products, however, were not specified). Approximately 1.5 to 3 log reductions in CFUs from original challenge densities were observed after 1 or 2 hours of incubation, corresponding to calculated MOI_{actual}s of approximately 3.5 to 7. The cultures subsequently resumed replication, though, to reach densities that were greater than starting bacterial densities after six hours post challenge. These recovering bacteria were not tested for phage resistance, but a population size of 10^3 bacteria would have to

have a very high per-capita mutation rate to consistently possess mutations to phage resistance. Instead, phage HY01 could be temperate, and the growing back bacteria therefore lysogens, though phage HY01's close relationship with the professionally lytic [62] phage T4 suggests that this alternative explanation for why such dramatic bacterial grow back could occur is unlikely. Phage densities were not monitored over time, so it is possible that rapid phage inactivation occurred, i.e., in the presence of cabbage, and that this hypothesized inactivation was followed by recovery of a small number of bacteria which by chance or due to their micro-location when originally treated did not encounter phages initially. Given the large number of phages applied, and the small number of bacteria, it is likely that passive treatment played a larger role than active treatment, though the slowness of *S. flexneri* reductions is at least suggestive that small microcolonies could have formed prior to eventual phage infection, thereby allowing for the possibility of locally active treatment.

1.12. Minh et al.

Minh et al. [63] treated raw chicken meat with the anti-*Escherichia coli* phage, EBL116, with a subsequent 5 ℃ or 25 ℃ incubation. A challenge of 3×10^4 CFU/chicken piece (a reported 4 cm^2) was followed with a phage dose of 3×10^7 PFU (phage density within the phage formulated product not specified). Reductions in CFUs from original challenge densities in excess of 1.5 log were observed after 2 hours of incubation. MOI_{input} was 10^3 while the observed reductions are consistent with MOI_{actual}s of less than 5, implying that most applied phages did not reach bacteria. Impact of greater phage doses was not determined though if all bacteria were equally reachable by phages then we can speculate that a phage dose of approximately 10^8 PFU, a three-fold increase, could have been sufficient to achieve an approximation of complete bacterial eradication (i.e., as achieving a predicted MOI_{actual} of about 12). Ideally experiments employing ten-fold greater numbers, i.e., 10^9 PFU, would have been attempted as a bacteria-killing positive control, and particularly so given approximately 3-log increases in bacterial densities post phage-associated reductions in bacterial densities at 25 ℃. Active treatment presumably could not occur at 5 ℃ and there is no evidence presented indicating that it may have occurred at 25 ℃, i.e., (i) there was no delay between bacterial challenge and phage application, (ii) reduction in bacterial numbers occurred over no more than a two-hour period, and (iii) no description of phage titers over the course of the experiment is provided. Presumably, therefore, what was observed was passive treatment.

1.13. Opez-Cuevas et al.

Opez-Cuevas et al. [64] treated tomato surfaces with the temperate, anti-*Salmonella enterica* Typhimurium phage, P22, with a subsequent 10 ℃ or 20 ℃ incubation. In preliminary experiments in broth (at 37 ℃) they found that application of approximately 10^9 phages per ml reduced bacterial densities from approximately 10^6 CFU/ml to undetectable levels. The application of approximately 10^8 phages per ml resulted instead in only slight declines in bacterial growth rate relative to the phage-free control. My guess is that the higher phage concentration resulted in abortive infections [65], or perhaps (but speculatively) lysis from without [66], while the lower phage concentration resulted predominantly in lysogenic cycles [67]. As reviewed in [67], however, application of phages in lower densities, resulting in lower MOI_{actual}s, results in

lower likelihoods of non-antibacterial lysogenic cycles and higher likelihoods of antibacterial lytic cycles. Furthermore, the phages employed displayed clear plaques which would be consistent with a relative rarity of display of lysogenic cycles, though not necessarily with the high phage MOIs likely seen given exposure to 10^8 phages per ml. In the actual biocontrol experiments, bacterial challenge appears to have consisted of immersion of bacteria into 10^9 CFU/ml for 15 min, resulting in between 10^6 and 10^7 adherent CFU per tomato. The surfaces of tomatoes appear to then have been dried for one hour. Subsequently tomatoes were sprayed for 45 seconds with 10^{11} PFU/ml "to simulate packinghouse spray disinfecting conditions." This dosing approach is to be commended, though it makes it difficult to estimate MOI_{input}. At 10 ℃ after 1 day there is approximately a 1 log reduction in bacterial densities with phages and no bacterial growth without phages, suggesting an MOI_{actual} between 2 and 3. At 20 ℃, surprisingly, reductions were somewhat less, though perhaps not surprisingly if (speculating) there is a favoring of lysogenic cycles over lytic cycles under these conditions. At 10 ℃, without phages, there was little evidence of bacterial growth, but declines in bacterial densities were seen after four days when phages were present, suggesting ongoing phage infections over a seven-day period. At 20 ℃ there was a small amount of bacterial growth absent phages and little phage impact over time, i.e., as could be consistent with phage display of lysogenic cycles. The application of high densities of phages, though at unknown volumes, in combination with little bacterial growth in the absence of phages, is suggestive of passive treatment. Though it is conceivable that locally active treatment occurred at 20 ℃, there is little evidence of it occurring to a substantial extent, and no evidence of globally active treatment since phage densities were not determined over time.

1.14. Seo et al.

Seo et al. [68] treated various meats with an anti-*Escherichia coli* O157:H7 phage, BPECO19, with a subsequent 4 ℃ or 37 ℃ incubation. A challenge of 10^5 CFU/cm^2 was spread on meat samples and followed by spreading of a phage dose of 10^8, 10^9, or 10^{10} PFU/cm^2 (the latter as based on indicated MOIs, i.e., MOI_{input}, of 10^3, 10^4, or 10^5; phage densities within the phage formulated products, however, were not specified). The study shows, with raw beef at 4 ℃, a remarkable progression as phage numbers were increased, with less than a 1 log reduction in CFUs from the original challenge densities after what appears to be less than 10 hours at the lowest phage density, about a 2-log reduction after less than 20 hours at the next highest phage density, and reductions all the way to zero in less than ten hours given the highest phage density (these correspond to estimated MOI_{actual}s of 2.3, 4.6, and greater than 10, respectively). At the two lower bacterial densities there was, approximately following the CFU minima, a gradual rise in bacterial numbers which paralleled that of the phage-less control. This increase in CFUs was perhaps due to separation of already replicated bacteria since our expectation is that replication of this bacterium in fact would not be occurring at this temperature (4 ℃). The implication is that phages reach bacteria early on and then either become inactivated or otherwise can no longer reach bacteria, the latter perhaps due to interference by the food matrix. In any case, these results represent an outstanding indication of both the benefits of supplying more phages and the ease with which such higher-phage-density trials can be introduced into studies. On the other hand, there was no appreciable time gap between the bacterial challenge and phage application. Thus, passive treatment unquestionably seems to be at

work here, that is, given the rapidity of bacterial inactivation, the high phage numbers applied (especially for the highest phage numbers), and the low temperature of incubation. Declines in bacterial densities with phage treatment apparently were *not* observed at 37 ℃ but with data not presented.

1.15. Snyder et al.

Snyder et al. [69] treated cut green pepper and baby spinach leaves with an anti-*Escherichia coli* O157:H7 phage, OSY-SP, with a subsequent 4 ℃ or 25 ℃ incubation. Bacterial challenge was described as follows, "Samples of fresh produce were spot-inoculated with 10-μl aliquots of the 12-h bacterial culture… Green pepper pieces were inoculated on cut-edge surfaces to facilitate absorption while spinach leaves were spot-inoculated on leaf surfaces with 10 × 10-μl spots per 50 g. Inocula were allowed to dry for 1 h, at ambient temperature, prior to treatment." Phage treatment consisted of rinsing using a 10^8 PFU/ml purified phage suspension, 5 min for green pepper and 2 min for spinach leaves. On spinach leaves at 25 ℃, reductions were approximately 2 log relative to rinsing without phages. This suggests an MOI_{actual} of about 5 (MOI_{input} cannot be easily determined for these experiments). In other experiments, reductions were also roughly 2 log relative to rinsing, also representing an MOI_{actual} of about 5. A doubling of phage densities potentially could have raised this MOI_{actual} to a preferred value of 10, though conservatively perhaps 10^9 PFU/ml would have been preferable to consistently reach this value under real-world conditions, or 10^{10} PFU/ml as a bacteria-killing positive control. In a manner, such increased phage dosing was attempted though via a less controlled means, that is, by initially incubating at 25 ℃ so as to encourage active treatment, and this may have made a small positive impact. Globally active treatment, however, is dependent on overall concentrations of contaminating bacteria, which are not under the control of food producers, suggesting that explicitly increasing phage densities within doses would be a preferable strategy towards increasing phage densities *in situ*, that is, rather than relying on globally active treatment. On the other hand, to the extent that locally active treatment was desired, then experiments explicitly testing for this utility would be needed, though it is not obvious that there would be utility to encouraging increases in numbers of contaminating bacteria solely for the sake of increasing phage densities *in situ*, especially given that phage densities instead may be relatively easily increased in terms of dosing. To the extent indicated, as bacteria killing was rapid and did not continue over the course of further incubation, plus was effective at 4 ℃, it likely was passive treatment that was occurring. Phage densities nevertheless increased over the first few hours following application, suggesting some potential for active treatment, though this occurred even at 4 ℃ so the mechanism of this increase is uncertain, plus there is no evidence that these increases resulted in increased bacteria killing. It is also possible bacteria replicated between challenge and treatment, and therefore that locally active treatment could to a degree have occurred, particularly given incubations at 25 ℃, though this is merely speculation.

1.16. Soffer et al.

Soffer et al. [70] treated cantaloupe, chicken, lettuce, sushi-grade tuna, and turkey trims with the anti-*Salmonella* phage formulated product, SalmoLyse®, with a subsequent room temperature

incubation. Challenges consisted of 1.25×10^3 CFU/g of a single *Salmonella* serotype applied by spreading to turkey trims, incubated at room temperature for one hour, and then treated with either 9×10^6 PFU/g (involving application of a 2×10^9 PFU/ml suspension) or 2×10^7 PFU/g (involving application of a 10^9 PFU/ml suspension). Phages were applied using an air-atomizer sprayer. After a 5 min delay, the trimmings were ground and then enumerated for CFUs. With the other foods, bacterial challenge was with three *Salmonella* strains: 5×10^2 CFU/g for lettuce; 1.5×10^3 CFU/g for chicken; and 2×10^3 CFU/g for cantaloupe and tuna. A 10^9 PFU/ml suspension was then applied at various volumes resulting in “Standard” treatments of 9×10^6 PFU/g, “Low” treatments of 4×10^6 PFU/g, and, for chicken, a “Very low” treatment of 2×10^6 PFU/g. Reductions in CFUs ranged from 0.5 to 1.1 logs (plus 0.4 logs with the “Very low” treatment of chicken). MOI_{input}s ranged from 2×10^3 to 2×10^4 versus calculated MOI_{actual}s ranging from about 1 to about 2.5. This suggests that “Standard” volumes probably, ideally, would have been approximately four times greater while bacteria-killing positive controls ideally would have been about 40 times greater. Unfortunately, however, it is questionable to what degree the application of such higher volumes would even be possible, i.e., in terms of avoiding phage losses, as a four-fold increase would be roughly 1 ml per 25 gram sample while forty times would be 10 ml per 25 gram sample. In addition, there was no statistically significant difference in levels of bacteria killing between the different volumes applied, so it is questionable that the application of greater volumes would have had much of an impact. This would be versus employing greater phage densities per volume, which could be a factor since a two-fold decrease in phage densities resulted in approximately a two-fold decrease in log killing in the case of treatment of turkey trims, even if this difference was not necessarily statistically significantly different. Alternatively, the duration of treatment may have been extended from the reported five min to longer time frames, e.g., 20 min, though presumably 200 min for a positive control would be excessive given the potential for remaining bacteria to replicate over such a long time frame. Treatment here clearly was passive as it occurred over such short time spans.

1.17. Sukumaran et al.

Sukumaran et al. [71] treated chicken breast fillets with the anti-*Salmonella* spp. phage formulated product, SalmoFresh™, with a subsequent 4 ℃ or room temperature incubation. A challenge of 100 µl was applied in an unspecified manner to 25 grams of meat, resulting in approximately 10^3 CFU/g. After 30 min at room temperature, the inoculated meat was then either dipped for 20 seconds in the phage formulated product or instead surface-treated (0.5 ml, also as applied in an unspecified manner). The phage dose, in either case, consisted of 10^9 PFU/ml. With both dipping and surface application, with incubation at 4 ℃, CFUs were reduced by less than 1 log on “Day 0” (which I assume means 2 hours) and by a little over 1 log on “Day 1” (which I assume means 24 hours; note, though, that bacterial counts declined without treatment by “Day 1” as well). These results suggest MOI_{actual}s roughly in the range of 2. For surface application, MOI_{input} appears to have been $(0.5 \times 10^9)/(25 \times 10^3)$, or 2×10^4. Given the similarity of outcomes in terms of reductions in bacterial numbers, we can assume that MOI_{input} may have been similar with dipping, implying that most applied phages did not reach bacteria. Impact of greater phage doses was not determined though if all bacteria were equally reachable by phages then we can speculate that a phage titer dose of approximately 4×10^9 PFU/ml, a four-fold increase, could have been sufficient to

achieve an approximation of complete bacterial eradication (i.e., as achieving a predicted MOI_{actual} of about 10), though ideally experiments employing ten-fold greater numbers, i.e., 4×10^{10} PFU/ml, would have been attempted as a bacteria-killing positive control. As in actuality the commercial formulated product consists of 10^{10} phages/ml, using longer dipping times may have utility in this case, though an alternative consideration is that suspension of virions in deionized water to produce the 10^9 PFU/ml formulated products—"Inoculated samples were immersed in 100mL of bacteriophage solution (10^9 PFU/mL) prepared in sterile DI water…"—could have resulted in declines in phage viability or, instead, in reductions in virion adsorption ability. The impact of surface phage treatment followed by room temperature incubation was similar over the short term (an initial 0.5 log reduction in CFUs), though after eight hours bacterial densities with phage treatment were approximately 0.5 log greater than the concentrations at which they had started. Clearly an achievement of far greater bacterial killing early on would have been preferable in this case, which potentially could have been achieved given application, for example, of greater phage numbers. No evidence of active treatment is otherwise apparent in this study suggesting that it was passive treatment which was observed.

1.18. Suggestions toward improving future research

In considering the articles quantitatively reviewed above, a number of generalizations seem clear:

First, research goals should be to prevent bacteria from exceeding predetermined maximum allowable numbers rather than just reducing numbers of bacteria relative to untreated controls. Especially, if there is a potential for bacteria to replicate to an appreciable extent post phage application, then it is should be obvious that sufficient phage numbers should be applied to reduce bacterial densities sufficiently that some post-treatment recovery in bacterial numbers will *not* result in their exceeding maximum allowable numbers. Ideally, therefore, unless bacteria otherwise are adequately prevented from replicating, then the number of surviving targeted bacteria post phage treatment should be sufficiently low that even with grow back bacterial numbers will remain sufficiently low. This, of course, also implies that bacterial challenges should consist of reasonably well expected numbers of target bacteria, and therefore that the number bacteria that phages both encounter upon experimental application and are expected to reduce in number is realistic. Sulakvelidze [30] provides a discussion of both the impact of different strategies of phage application and resulting allowable numbers of remaining bacteria, though also provides a caveat, from p. 3144: "assuming that those laboratory data are reproducible in commercial food-processing facilities".

Second, even if bacteria cannot replicate following phage application, then goals of experiments should be to reduce bacterial numbers not just to levels which are considered to be safe but to somewhat lower numbers assuming that ideal conditions as may be approximated in the laboratory will not be seen in the real world. A corollary to these first two points (this and the previous paragraph), from [5], p. 93, is that "bacteriophage treatments are not a 'magic bullet' and that they should be used in the hurdle approach to food safety." For similar statements, see [10,11]. That is, if phage application is not sufficient to adequately reduce bacterial numbers or to prevent the replication of bacteria to unacceptable levels, then one or more additional mechanisms ("hurdles")

must be in place to augment phage activity, e.g., such as refrigeration. Nonetheless, overall goals in the laboratory should be to reduce bacterial numbers not just to acceptable levels but to better than acceptable levels and then, upon scale-up, reductions perhaps to simply acceptable levels should be the minimum goal.

Third, greater effort needs to be made to design experiments which are better approximations of what might be observed outside of the laboratory. Thus, from [17], p. 66: "As a general rule, the most indicative studies will be those where realistic contamination and treatment scenarios are used." Particularly, realistically long delays between bacterial challenge and phage administration should be employed in the case of foods that tend to be contaminated well prior to their arrival for phage treatment. Alternatively, if phages are applied at the point of anticipated contamination, e.g., such that they need to be mixed into semi-solid foods, then degrees of mixing should approximate what is expected industrially rather than necessarily as may be maximized in the laboratory. A corollary to this latter point, also from [5], p. 98, is that "None of the studies had evaluated the use of phage in the field either in pilot facilities or in the factory environment." Exceptional as reviewed here, however, is the study by Kurtböke et al. [59], which studied reductions in naturally acquired bacterial populations. A related issue is that of growth of bacterial cultures prior to challenge under substantially different conditions, in terms of temperature and nutrients, than as may be found while in association with foods, as well as bacterial challenges using stationary phase cultures. These are all factors which could affect phage infections given phage application relatively soon after bacterial challenge, that is, before bacteria have completely adapted to the food environment. Thus, longer delays between challenge and treatment, e.g., many hours or even days, could result in bacteria which are better physiologically adapted to the conditions in or on foods, and thereby may serve as more realistic phage targets.

Four, the questions of whether bacteria really are able to hide from phages within food matrix, or whether phage titers are increasing or decreasing over time *in situ*, or indeed whether the addition of more phages will result in greater levels of reduction in numbers of target bacteria need to be systematically explored. Such exploration should be done on a study-by-study basis especially for the latter issue of a relative lack of repeating of experiments using more phages when bacteria persist within foods after phage treatment when using fewer phages. Alternatively, justifications for not determining whether applying more phages will increase levels of bacteria killing—reasons including due to testing of commercially available preparations, or due to economics or technical issues—need to be explicitly stated within studies. A related issue is the question of whether formulated products contain expected numbers of phages, such as following diluting.

Five, research driven by statistically significant differences rather than more meaningful reductions in bacterial densities can be a serious hindrance to progression of the field. A different perspective on the same point is that solely statistically significant differences at best should be viewed as proof-of-principle results unless levels of reduction in bacterial densities are explicitly justified as real-world meaningful. A corollary is that one should assume that laboratory procedures both can and are optimized in a way that likely is difficult or impossible to scale up commercially—if nothing else, genetic differences likely will exist among target bacteria between those used in the laboratory and those found to have contaminated foods [35]—meaning that barely effective treatments in the laboratory are unlikely to be even barely effective in the factory. Instead, study goals should be both predetermined and explicitly stated while study end points should generally be

of substantially greater effectiveness than is expected to be necessary, e.g., 1 log overkill of targeted bacteria beyond acceptable numbers.

Six, measurements of phage dosing need to better approximate the manner in which phage dosing would be measured industrially, which generally will be in terms of phage concentrations within phage formulated products, that is, as phage titers, but also in combination with a reporting of the volumes of phage formulated product that are added to a given quantity of food. This is rather than per cm^2 measures unless similar dosing measures in fact are anticipated in the factory. Certainly multiplicity of infection in any sense of that term should not be relied upon since MOI_{input} is misleading and neither MOI_{input} nor MOI_{actual} are likely to ever be employed commercially as dosing measures.

Seven, it is possible to use calculations of bacterial survival to estimate actual multiplicities of infection achieved during treatments (MOI_{actual}), versus the ratio of phages applied to bacteria present (MOI_{input}). It can be helpful to make such calculations if only towards gaining a better appreciation of how efficiently phages are reaching bacteria [46], e.g., MOI_{input} of 10,000 versus MOI_{actual} of 8.5 [58]. Keeping track of phage titers present *in situ* over the course of treatments can be similarly helpful.

Eight, tighter enumeration time courses especially at the beginning of treatments, i.e., more time points, and particularly when bacteria are able to replicate absent phage presence, would allow for better appreciation of the dynamics of phage-bacterial interactions in the course of treatment. It is helpful, in other words, to know whether a majority of bacterial eradication is occurring over the first minutes or hours of treatment versus over the first day, even if goals are ones of multi-day suppression of bacterial counts.

Nine, especially when employing high phage doses, it is important in terms of assuring the validity of a study to explicitly demonstrate that bacteria are not being phage adsorbed in the course of bacterial enumeration. This can be accomplished through a combination of diluting bacteria prior to homogenization, employing phage- but not bacteria-inactivating virucides in the course of diluting and homogenizing, and seeding with marked bacteria in the course of enumeration steps to experimentally assure that bacterial numbers are not being reduced during enumeration procedures. This point was not otherwise emphasized within this Appendix, as researchers in fact do tend to dilute prior to homogenizing, but it is important to appreciate that the validity of a study rests wholly on the question of whether phage impact occurs prior to versus during bacterial enumeration, while assurance that the former (prior impact) is the case is difficult to simply infer.

Ten, and returning to the emphasis of the main text of this study, to the extent that microcolonies or bacterial arrangements in fact are present among contaminating bacteria within foods, and that such existence as clonal bacterial clumps is relevant to treatment success, then their presence in foods prior to phage application needs to be explicitly considered, perhaps particularly in terms of experimental design. Near-simultaneous application of challenge bacteria and treatment phages should result, however, in a low potential for bacteria to replicate into clumps prior to treatment. Thus, if the potential for phages to propagate within bacterial clumps in fact is an issue that is relevant to phage-mediated biocontrol of bacteria contaminating foods, then at least among the articles reviewed in this Appendix we are doing a relatively poor job of distinguishing among phages or treatment approaches in terms of this ability.

References

1. Abedon ST (2017) Information phage therapy research should report. *Pharmaceuticals (Basel)* 10: 43.
2. Abedon ST (2011) *Bacteriophages and Biofilms: Ecology, Phage Therapy, Plaques*, Hauppauge, New York: Nova Science Publishers.
3. Abedon ST, Alves DR, Phage therapy bibliography, 2017. Available from: http://publications.phage-therapy.org.
4. Alves DR, Abedon ST (2017) An online phage therapy bibliography: separating under-indexed wheat from overly indexed chaff. *AIMS Microbiol* 3: 525–528.
5. Food Standards Agency, Systematic and critical review on the potential of bacteriophages on foods, 2016. Available from: https://www.food.gov.uk/science/research/chemical-safety-research/additives-research/additives-general/fs102079.
6. Goodridge L, Abedon ST (2003) Bacteriophage biocontrol and bioprocessing: application of phage therapy to industry. *SIM News* 53: 254–262.
7. Hudson JA, Billington C, Carey-Smith G, et al. (2005) Bacteriophages as biocontrol agents in food. *J Food Prot* 68: 426–437.
8. Sulakvelidze A, Barrow P (2005) Phage therapy in animals and agribusiness, In: Kutter E, Sulakvelidze A, Editors, *Bacteriophages: Biology and Application*, Boca Raton, Florida: CRC Press, 335–380.
9. Hudson JA, Billington C, Bigwood T (2006) Phage inactivation of foodborne bacteria. *Food Australia* 58: 593–595.
10. Rees CED, Dodd CER (2006) Phage for rapid detection and control of bacterial pathogens in food. *Adv Appl Microbiol* 59: 159–186.
11. Hagens S, Loessner MJ (2007) Application of bacteriophages for detection and control of foodborne pathogens. *Appl Microbiol Biotechnol* 76: 513–519.
12. Strauch E, Hammerl JA, Hertwig S (2007) Bacteriophages: new tools for safer food? *J Consum Protect Food Safety* 2: 138–143.
13. García PB, Martínez B, Obeso JM, et al. (2008) Bacteriophages and their application in food safety. *Lett Appl Microbiol* 47: 479–485.
14. Goodridge LD (2008) Phages, bacteria, and food, In: Abedon ST, Editor, *Bacteriophage Ecology*, Cambridge, UK: Cambridge University Press, 302–331.
15. Hagens S, Offerhaus MI (2008) Bacteriophages, New Weapons for Food Safety. *Food Technol* 62: 46–54.
16. Atterbury RJ (2009) Bacteriophage biocontrol in animals and meat products. *Microb Biotechnol* 2: 601–612.
17. Hagens S, Loessner MJ (2010) Bacteriophage for biocontrol of foodborne pathogens: calculations and considerations. *Curr Pharm Biotechnol* 11: 58–68.
18. Coffey B, Mills S, Coffey A, et al. (2010) Phage and their lysins as biocontrol agents for food safety applications. *Annu Rev Food Sci Technol* 1: 449–468.
19. Gill JJ (2010) Practical and theoretical considerations for the use of bacteriophages in food systems, In: Sabour PM, Griffiths MW, Editors, *Bacteriophages in the Control of Food- and Waterborne Pathogens*, Washington, DC: ASM Press, 217–235.

20. García P, Rodríguez L, Rodríguez A, et al. (2010) Food biopreservation: promising strategies using bacteriocins, bacteriophages and endolysins. *Trends Food Sci Technol* 21: 373–382.
21. Hudson JA, McIntyre L, Billington C (2010) Application of bacteriophages to control pathogenic and spoilage bacteria in food processing and distribution, In: Sabour PM, Griffiths MW, Editors, *Bacteriophages in the Control of Food- and Waterborne Pathogens*, Washington, DC: ASM Press, 119–135.
22. Fieseler L, Loessner MJ (2011) Bacteriophages and food safety, In: Lacroix C, Editor, *Protective cultures, antimicrobial metabolites and bacteriophages for food and beverage biopreservation*, Cambridge, UK: Woodhead Publishing, 161–178.
23. Goodridge LD, Bisha B (2011) Phage-based biocontrol strategies to reduce foodborne pathogens in foods. *Bacteriophage* 1: 130–137.
24. Mahony J, McAuliffe O, Ross RP, et al. (2011) Bacteriophages as biocontrol agents of food pathogens. *Curr Opin Biotechnol* 22: 157–163.
25. Brovko LY, Anany H, Griffiths MW (2012) Bacteriophages for detection and control of bacterial pathogens in food and food-processing environment. *Adv Food Nutr Res* 67: 241–288.
26. García P, Martínez B, Rodríguez L, et al. (2012) Bacteriophages as biocontrol agents in dairy processing, In: Quiberoni A, Reinheimer J, Editors, *Bacteriophages in Dairy Processing*, Hauppauge, New York: Nova Science Publishers, 269–294.
27. Niu YD, Stanford K, McAllister TA, et al. (2012) Role of phages in control of bacterial pathogens in food, In: Hyman P, Abedon ST, Editors, *Bacteriophages in Health and Disease*, Wallingford, UK: CABI Press, 240–255.
28. Sillankorva SM, Oliveira H, Azeredo J (2012) Bacteriophages and their role in food safety. *Int J Microbiol* 2012: 863945.
29. Klumpp J, Loessner MJ (2013) New research on bacteriophages and food safety, In: Sofos J, Editor, *Advances in Microbial Food Safety*, 16 Eds., Elsevier, 321–339.
30. Sulakvelidze A (2013) Using lytic bacteriophages to eliminate or significantly reduce contamination of food by foodborne bacterial pathogens. *J Sci Food Agric* 93: 3137–3146.
31. Anany H, Brovko LY, El-Arabi T, et al. (2014) Bacteriophages as antimicrobials in food products: history, biology and application. *Handbook of Natural Antimicrobials for Food Safety and Quality* 69.
32. Endersen L, O'Mahony J, Hill C, et al. (2014) Phage therapy in the food industry. *Annu Rev Food Sci Technol* 5: 327–349.
33. Perez PR, Grande BMJ, Galvez A, et al. (2015) Application of bacteriophages in post-harvest control of human pathogenic and food spoiling bacteria. *Crit Rev Biotechnol* 14: 1–11.
34. Petsong K, Vongkamjan K (2015) Applications of *Salmonella* bacteriophages in the food production chain, In: *The Battle Against Microbial Pathogens: Basic Science, Technology Advances and Educational Programs*, 275–283.
35. Strydom A, Witthuhn CR (2015) *Listeria monocytogenes*: a target for bacteriophage biocontrol. *Compr Rev Food Sci Food Saf* 14: 694–704.
36. Zaczek M, Weber-Dabrowska B, Górski A (2015) Phages in the global fruit and vegetable industry. *J Appl Microbiol* 118: 537–556.
37. Bai J, Kim YT, Ryu S, et al. (2016) Biocontrol and rapid detection of food-borne pathogens using bacteriophages and endolysins. *Front Microbiol* 7: 474.

38. Cooper IR (2016) A review of current methods using bacteriophages in live animals, food and animal products intended for human consumption. *J Microbiol Meth* 130: 38–47.
39. Kazi M, Annapure US (2016) Bacteriophage biocontrol of foodborne pathogens. *J Food Sci Technol* 53: 1355–1362.
40. Pérez PR, Grande BMJ, Gálvez A, et al. (2016) Application of bacteriophages in post-harvest control of human pathogenic and food spoiling bacteria. *Crit Rev Biotechnol* 36: 851–861.
41. Pietracha D, Misiewicz A (2016) Use of products containing a phage in food Industry as a new method for *Listeria monocytogenes* elimination from food (*Listeria monocytogenes* phages in food industry)—a review. *Czech J Food Sci* 34: 1–8.
42. Hussain MA, Liu H, Wang Q, et al. (2017) Use of encapsulated bacteriophages to enhance farm to fork food safety. *Crit Rev Food Sci Nutr* 57: 2801–2810.
43. Sabouri S, Sepehrizadeh Z, Amirpour-Rostami S, et al. (2017) A minireview on the *in vitro* and *in vivo* experiments with anti-*Escherichia coli* O157:H7 phages as potential biocontrol and phage therapy agents. *Int J Food Microbiol* 243: 52–57.
44. Abedon ST (2016) Phage therapy dosing: the problem(s) with multiplicity of infection (MOI). *Bacteriophage* 6: e1220348.
45. Kasman LM, Kasman A, Westwater C, et al. (2002) Overcoming the phage replication threshold: a mathematical model with implications for phage therapy. *J Virol* 76: 5557–5564.
46. Abedon ST, Expected efficacy: applying killing titer estimations to phage therapy experiments, 2017. Available from: http://killingtiter.phage-therapy.org.
47. Abedon ST, Killing titer calculator, 2017. Available from: http://killingtiter.phage-therapy.org/calculator.html.
48. Abedon ST, Multiplicity of infection calculator, 2017. Available from: http://moicalculator.phage.org.
49. Baños A, García-López JD, Núñez C, et al. (2016) Biocontrol of *Listeria monocytogenes* in fish by enterocin AS-48 and *Listeria* lytic bacteriophage P100. *LWT-Food Sci Technol* 66: 672–677.
50. Boyacioglu O, Sulakvelidze A, Sharma M, et al. (2016) Effect of a bacteriophage cocktail in combination with modified atmosphere packaging in controlling *Listeria monocytogenes* on fresh-cut spinach. *Irish J Agr Food Res* 55: 74–79.
51. El HL, Roy JP, Khalil GE, et al. (2016) Efficacy of two *Staphylococcus aureus* phage cocktails in cheese production. *Int J Food Microbiol* 217: 7–13.
52. Galarce N, Escobar B, Rojas V, et al. (2016) Application of a virulent bacteriophage cocktail leads to reduction of *Salmonella enterica* serovar Enteritidis counts in processed meat products. *Biocont Sci Technol* 26: 462–475.
53. Abedon ST (2016) Phage therapy dosing: The problem(s) with multiplicity of infection (MOI). *Bacteriophage* 6: e1220348.
54. Gencay YE, Ayaz ND, Copuroglu G, et al. (2016) Biocontrol of Shiga toxigenic *Escherichia coli* O157: H7 in Turkish raw meatball by bacteriophage. *J Food Saf* 36: 120–131.
55. Hong Y, Schmidt K, Marks D, et al. (2016) Treatment of *Salmonella*-contaminated eggs and pork with a broad-spectrum, single bacteriophage: assessment of efficacy and resistance development. *Foodborne Pathog Dis* 13: 679–688.
56. Hudson J, Billington C, Premaratne A, et al. (2016) Inactivation of *Escherichia coli* O157:H7 using ultraviolet light-treated bacteriophages. *Food Sci Technol Int* 22: 3–9.

57. Carlson K (2005) Working with bacteriophages: common techniques and methodological approaches, In: Kutter E, Sulakvelidze A, Editors, *Bacteriophages: Biology and Application*, Boca Raton, Florida: CRC Press, 437–494.
58. Iacumin L, Manzano M, Comi G (2016) Phage inactivation of *Listeria monocytogenes* on San Daniele dry-cured ham and elimination of biofilms from equipment and working environments. *Microorganisms* 4: 4.
59. Kurtboke DI, Palk A, Marker A, et al. (2016) Isolation and characterization of *Enterobacteriaceae* species infesting post-harvest strawberries and their biological control using bacteriophages. *Appl Microbiol Biotechnol* 100: 8593–8606.
60. Ladero V, Gómez-Sordo C, Sanchez-LLana E, et al. (2016) Q69 (an *E. faecalis*-Infecting bacteriophage) as a biocontrol agent for reducing tyramine in dairy products. *Front Microbiol* 7: 445.
61. Lee H, Ku HJ, Lee DH, et al. (2016) Characterization and genomic study of the novel bacteriophage HY01 infecting both *Escherichia coli* O157:H7 and *Shigella flexneri*: potential as a biocontrol agent in food. *PLoS One* 11: e0168985.
62. Hobbs Z, Abedon ST (2016) Diversity of phage infection types and associated terminology: the problem with 'Lytic or lysogenic'. *FEMS Microbiol Lett* 363.
63. Minh DH, Minh SH, Honjoh Ki, et al. (2016) Isolation and bio-control of Extended Spectrum Beta-Lactamase (ESBL)-producing *Escherichia coli* contamination in raw chicken meat by using lytic bacteriophages. *Food Sci Technol* 71: 339–346.
64. Opez-Cuevas O, Castro-del Campo N, Ramirez K, et al. (2016) Biocontrol of *Salmonella* Typhimurium growth in tomato surface by bacteriophage P22. *Afr J Microbiol Res* 10: 528–534.
65. Molineux IJ (2006) Fifty-three years since Hershey and Chase; much ado about pressure but which pressure is it? *Virology* 344: 221–229.
66. Abedon ST (2011) Lysis from without. *Bacteriophage* 1: 46–49.
67. Abedon ST (2017) Commentary: Communication between viruses guides lysis-ysogeny decisions. *Front Microbiol* 8: 983.
68. Seo J, Seo DJ, Oh H, et al. (2016) Inhibiting the growth of *Escherichia coli* O157:H7 in beef, pork, and chicken meat using a bacteriophage. *Korean J Food Sci Anim Resour* 36: 186–193.
69. Snyder AB, Perry JJ, Yousef AE (2016) Developing and optimizing bacteriophage treatment to control enterohemorrhagic *Escherichia coli* on fresh produce. *Int J Food Microbiol* 236: 90–97.
70. Soffer N, Abuladze T, Woolston J, et al. (2016) Bacteriophages safely reduce *Salmonella* contamination in pet food and raw pet food ingredients. *Bacteriophage* 6: e1220347.
71. Sukumaran AT, Nannapaneni R, Kiess A, et al. (2016) Reduction of *Salmonella* on chicken breast fillets stored under aerobic or modified atmosphere packaging by the application of lytic bacteriophage preparation SalmoFresh^{TM}. *Poult Sci* 95: 668–675.

AIMS Press

© 2017 Stephen T. Abedon, licensee AIMS Press. This is an open access article distributed under the terms of the Creative Commons Attribution License (http://creativecommons.org/licenses/by/4.0)
